# Comparing the 5-Year Diabetes Outcomes of Sleeve Gastrectomy and Gastric Bypass: The National Patient-Centered Clinical Research Network (PCORNet) Bariatric Study

**DOI:** 10.1001/jamasurg.2020.0087

**Published:** 2020-03-04

**Authors:** Kathleen M. McTigue, Robert Wellman, Elizabeth Nauman, Jane Anau, R. Yates Coley, Alberto Odor, Julie Tice, Karen J. Coleman, Anita Courcoulas, Roy E. Pardee, Sengwee Toh, Cheri D. Janning, Neely Williams, Andrea Cook, Jessica L. Sturtevant, Casie Horgan, David Arterburn

**Affiliations:** 1 Department of Medicine, University of Pittsburgh, Pittsburgh, Pennsylvania; 2 Department of Epidemiology, University of Pittsburgh, Pittsburgh, Pennsylvania; 3 Kaiser Permanente Washington Health Research Institute, Seattle; 4 Louisiana Public Health Institute, New Orleans; 5 Center for Health Technology, University of California, Davis, Davis; 6 PaTH Clinical Data Research Network, Pennsylvania State University, Hershey; 7 Department of Research and Evaluation, Kaiser Permanente Southern California, Pasadena; 8 Department of Surgery, University of Pittsburgh, Pittsburgh, Pennsylvania; 9 Department of Population Medicine, Harvard Medical School, Harvard Pilgrim Health Care Institute, Boston, Massachusetts; 10 Duke Clinical & Translational Science Institute, Durham, North Carolina; 11 Mid-South Clinical Data Research Network, Meharry-Vanderbilt Alliance Community Partner, Nashville, Tennessee; 12 Now with Community Partners Network Inc, Nashville, Tennessee

## Abstract

**Importance:**

Bariatric surgery can lead to substantial improvements in type 2 diabetes (T2DM), but outcomes vary across procedures and populations. It is unclear which bariatric procedure has the most benefits for patients with T2DM.

**Objective:**

To evaluate associations of bariatric surgery with T2DM outcomes.

**Design, Setting, and Participants:**

This cohort study was conducted in 34 US health system sites in the National Patient-Centered Clinical Research Network Bariatric Study. Adult patients with T2DM who had bariatric surgery between January 1, 2005, and September 30, 2015, were included. Data analysis was conducted from April 2017 to August 2019.

**Interventions:**

Roux-en-Y gastric bypass (RYGB) or sleeve gastrectomy (SG).

**Main Outcome and Measures:**

Type 2 diabetes remission, T2DM relapse, percentage of total weight lost, and change in glycosylated hemoglobin (hemoglobin A_1c_).

**Results:**

A total of 9710 patients were included (median [interquartile range] follow-up time, 2.7 [2.9] years; 7051 female patients [72.6%]; mean [SD] age, 49.8 [10.5] years; mean [SD] BMI, 49.0 [8.4]; 6040 white patients [72.2%]). Weight loss was significantly greater with RYGB than SG at 1 year (mean difference, 6.3 [95% CI, 5.8-6.7] percentage points) and 5 years (mean difference, 8.1 [95% CI, 6.6-9.6] percentage points). The T2DM remission rate was approximately 10% higher in patients who had RYGB (hazard ratio, 1.10 [95% CI, 1.04-1.16]) than those who had SG. Estimated adjusted cumulative T2DM remission rates for patients who had RYGB and SG were 59.2% (95% CI, 57.7%-60.7%) and 55.9% (95% CI, 53.9%-57.9%), respectively, at 1 year and 86.1% (95% CI, 84.7%-87.3%) and 83.5% (95% CI, 81.6%-85.1%) at 5 years postsurgery. Among 6141 patients who experienced T2DM remission, the subsequent T2DM relapse rate was lower for those who had RYGB than those who had SG (hazard ratio, 0.75 [95% CI, 0.67-0.84]). Estimated relapse rates for those who had RYGB and SG were 8.4% (95% CI, 7.4%-9.3%) and 11.0% (95% CI, 9.6%-12.4%) at 1 year and 33.1% (95% CI, 29.6%-36.5%) and 41.6% (95% CI, 36.8%-46.1%) at 5 years after surgery. At 5 years, compared with baseline, hemoglobin A_1c_ was reduced 0.45 (95% CI, 0.27-0.63) percentage points more for patients who had RYGB vs patients who had SG.

**Conclusions and Relevance:**

In this large multicenter study, patients who had RYGB had greater weight loss, a slightly higher T2DM remission rate, less T2DM relapse, and better long-term glycemic control compared with those who had SG. These findings can help inform patient-centered surgical decision-making.

## Key Points

**Question:**

How do type 2 diabetes (T2DM) outcomes compare across the 2 most common bariatric procedures?

**Findings:**

In this cohort study of 9710 adults with T2DM who underwent bariatric surgery, most patients who had Roux-en-Y gastric bypass or sleeve gastrectomy experienced T2DM remission at some point over 5 years of follow-up. Patients who had Roux-en-Y gastric bypass showed slightly higher T2DM remission rates, better glycemic control, and fewer T2DM relapse events than patients who had sleeve gastrectomy.

**Meaning:**

Understanding diabetes outcomes of different bariatric procedures will help surgeons and patients with diabetes make informed health care choices.

## Teaser

This cohort study evaluates associations of sleeve gastrectomy and Roux-en-Y gastric bypass bariatric surgery with type 2 diabetes outcomes in adult patients.

## Introduction

Bariatric surgery appears more effective than medical care alone for improving diabetes outcomes.^1,2,3^ Remission of type 2 diabetes (T2DM) is common after bariatric surgery^4,5,6,7^ and may reduce risk for subsequent microvascular and macrovascular disease.^8,9,10,11^ However, T2DM remission rates after bariatric surgery vary substantially across procedures and populations^4,5,6,7^ and T2DM relapse has been reported in approximately a quarter to half of patients who have bariatric surgery and achieve remission.^6,7,12^

Studies focusing on the 2 most common bariatric procedures, sleeve gastrectomy (SG) and Roux-en-Y gastric bypass (RYGB), show mixed evidence in terms of T2DM outcomes, especially in the longer term.^13,14,15,16,17,18,19,20,21,22^ It is unclear how the choice between them is likely to affect T2DM. The comparison is particularly salient because SG has begun to supplant RYGB as the dominant bariatric procedure over the past decade, despite limited long-term comparative data.^23,24,25^

The PCORnet Bariatric Study (PBS),^25,26^ one of the first scientific initiatives of PCORnet, the National Patient-Centered Clinical Research Network,^27,28^ was designed to examine the effectiveness of common bariatric procedures. This article compares T2DM outcomes in PCORnet up to 5 years following surgery for patients who had SG or RYGB. Secondary analyses assess the procedures’ outcomes on body weight and glycemic control independent of diabetes remission.

## Methods

### Cohort Identification

The PBS cohort was previously described.^25^ Patients in the T2DM analyses underwent a primary bariatric procedure at 34 PCORnet-affiliated health systems (eTable 1 in the Supplement) from January 1, 2005, through September 30, 2015. Procedures were identified from more than 59 million patient records using *the International Classification of Diseases, Ninth Revision, Clinical Modification* (*ICD-9-CM*), *Current Procedure Terminology* version 4, and Healthcare Common Procedure Coding System codes. We defined patients with diabetes as having a hemoglobin A_1c_ (HbA_1c_) level of 6.5% or more or a T2DM medication prescription in the year before surgery. Patients taking only metformin, thiazolidinedione, or liraglutide needed an *ICD-9-CM* or Systematized Nomenclature of Medicine (SNOMED) code for T2DM or an HbA_1c_ level of 6.5% or more in the year before surgery to be eligible for inclusion. We excluded patients 80 years or older, those without T2DM, and individuals without relevant outcomes data (eFigure 1 and eAppendix 1 in the Supplement).

The Kaiser Permanente Washington Health Research Institute obtained institutional review board approval for oversight of data collection and analyses. Participating sites obtained approval or formal determination that these analyses was not human subjects research.^25^ A waiver of Health Insurance Portability and Privacy Act privacy authorization (and thus informed consent) was obtained for these analyses of deidentified data.

### Data Extraction

The PCORnet sites store standardized electronic health record data and sometimes other data (eg, claims data), in PCORnet datamarts.^28^ Programming queries from the PCORnet Coordinating Center extracted relevant deidentified data on eligible individuals from participating sites’ datamarts. Race/ethnicity, as recorded in electronic health records, was included, reflecting stakeholder input. Data were transmitted to the coordinating site for analysis. Codes from the *ICD-9-CM* and SNOMED identified diagnoses.

### Outcome Definitions

Remission from T2DM was defined as the first postsurgical occurrence of an HbA_1c_ level less than 6.5% (to convert to proportion of total hemoglobin, multiply by 0.04-0.07) following at least 6 months (presurgical and/or postsurgical time) without T2DM medication prescription orders. This HbA_1c_ level corresponds to a published, putative partial-remission threshold.^29^ It was identified by our clinical stakeholders as more clinically meaningful than the affiliated complete remission threshold (a normal hemoglobin A_1c_ level^29^ of <5.7%^30^), since an HbA_1c_ level less than 6.5% corresponds to a T2DM diagnosis.^30^ The occurrence of levels of 6.5% or more and/or a prescription for T2DM medication after remission defined relapse. The absolute change in HbA_1c_ level at 1 year, 3 years, and 5 years after surgery was calculated. The total weight loss percentage was estimated as (weight at surgery − weight at a postoperative point)/weight at surgery × 100).

### Statistical Analyses

We compared the associations of RYGB and SG with time to diabetes remission. Pairwise analyses were restricted to sites with at least 1 patient of each procedure type at each point. Possible confounding was addressed with direct adjustment for specific factors and deciles of an estimated propensity score. Analyses examining the adjustable gastric band procedure are provided in eAppendix 2 in the Supplement.

#### Primary Analysis

Cox proportional hazards models calculated the adjusted hazard ratio (HR) for remission and estimated the adjusted cumulative proportion of individuals remitting at 1 year, 3 years, and 5 years following surgery. The proportional hazards assumption was tested by including an interaction between time and bariatric surgery group in the model, then inspecting Schoenfeld residuals over time. Models were adjusted for predetermined baseline covariates: age, sex, race, Hispanic ethnicity, body mass index category (BMI; calculated as weight in kilograms divided by height in meters squared), HbA_1c_ category, Charlson/Elixhauser comorbidity score (range: −2 to 20; a higher score generally indicates worse health),^31^ the health conditions listed in Table 1, the number of diabetes medications, the number of days hospitalized in the year before surgery, the year of surgery, and the site of surgery.

**Table 1.** Sample Description of Adults Prior to Bariatric Surgery

Characteristic	No. (%)			Standardized Difference
	Roux-en-Y Gastric Bypass	Sleeve Gastrectomy	Overall	
Patients	6233 (64.2)	3477 (35.8)	9710 (100.0)	NA
Follow-up time, y				
Mean (SD)	3.3 (2.1)	2.2 (1.4)	2.9 (1.9)	NA
Median (IQR) [range]	3.2 (1.55-4.64) [0.01-10.7]	2.0 (0.99-3.26) [0.01-7.2]	2.7 (1.26-4.19) [0.01-10.7]	NA
Female	4576 (73.4)	2475 (71.2)	7051 (72.6)	0.05
Age, mean (SD), y	49.9 (10.4)	49.7 (10.8)	49.8 (10.5)	0.01
Age category, y				
20-44	1929 (31.0)	1117 (32.1)	3046 (31.4)	0.04
45-64	3819 (61.3)	2065 (59.4)	5884 (60.6)	
65-80	485 (7.8)	295 (8.5)	780 (8.0)	
BMI, mean (SD)	49.0 (8.2)	49.0 (8.6)	49.0 (8.4)	0.01
BMI category				
35-39	638 (10.2)	386 (11.1)	1024 (10.6)	0.06
40-49	3250 (52.1)	1781 (51.2)	5031 (51.8)	
50-59	1739 (27.9)	917 (26.4)	2656 (27.4)	
≥60	606 (9.7)	393 (11.3)	999 (10.3)	
Weight, mean (SD), kg	125.6 (25.6)	125.6 (27.1)	125.63 (26.1)	0.00
Weight, kg				
45.4-90	253 (4.1)	165 (4.8)	418 (4.3)	0.06
90-135	4025 (64.6)	2238 (64.4)	6263 (64.6)	
135-180	1743 (28.0)	927 (26.7)	2670 (27.5)	
180-225	187 (3.0)	132 (3.8)	319 (3.3)	
225-275	20 (0.3)	11 (0.3)	31 (0.3)	
Missing	5 (0.1)	4 (0.1)	9 (0.1)	
Year or year range of surgery				
2005-2009	969 (15.6)	53 (1.5)	1022 (10.5)	0.75
2010	1049 (16.8)	216 (6.2)	1265 (13.0)	
2011	1250 (20.1)	570 (16.4)	1820 (18.7)	
2012	1037 (16.6)	657 (18.9)	1694 (17.5)	
2013	798 (12.8)	743 (21.4)	1541 (15.9)	
2014	744 (11.9)	840 (24.2)	1584 (16.3)	
2015	386 (6.2)	398 (11.5)	784 (8.1)	
Hispanic ethnicity	1407 (22.9)	971 (28.3)	2378 (24.8)	0.12
Missing	91 (1.5)	42 (1.2)	133 (1.4)	
Race				
Asian	86 (1.6)	69 (2.4)	155 (1.9)	0.28
African American	900 (16.6)	800 (27.3)	1700 (20.3)	
Multiple	3 (0.1)	5 (0.2)	8 (0.1)	
White	4136 (76.2)	1904 (64.9)	6040 (72.2)	
Pacific Islander	32 (0.6)	19 (0.7)	51 (0.6)	
Native American	49 (0.9)	21 (0.7)	70 (0.8)	
Other	225 (4.1)	117 (4.0)	342 (4.1)	
Missing overall	802 (12.9)	542 (15.6)	1344 (13.8)	
Hemoglobin A_1c_, mean (SD)	7.3 (1.3)	7.1 (1.2)	7.2 (1.3)	0.17
Hemoglobin A_1c_ category, %				
<6.5	1554 (24.9)	922 (26.5)	2476 (25.5)	0.19
6.5-6.9	1408 (22.6)	951 (27.4)	2359 (24.3)	
7.0-7.9	1738 (27.9)	995 (28.6)	2733 (28.2)	
8.0-8.9	834 (13.4)	354 (10.2)	1188 (12.2)	
≥9.0	699 (11.2)	255 (7.3)	954 (9.8)	
Total diabetes medications, mean (SD), No.	1.70 (1.1)	1.60 (1.1)	1.66 (1.1)	0.09
Total diabetes medications, No.				
0	1096 (17.6)	747 (21.5)	1843 (19.0)	0.11
1	1354 (21.7)	772 (22.2)	2126 (21.9)	
2	2447 (39.3)	1266 (36.4)	3713 (38.2)	
3	1048 (16.8)	546 (15.7)	1594 (16.4)	
4-7	288 (4.6)	146 (4.2)	434 (4.5)	
Diabetes medications				
Biguanides	4109 (65.9)	2237 (64.3)	6346 (65.4)	0.03
*GLP-1* receptor agonists	278 (4.5)	148 (4.3)	426 (4.4)	0.01
Insulins	3047 (48.9)	1645 (47.3)	4692 (48.3)	0.03
Sulfonylureas	2054 (33.0)	1058 (30.4)	3112 (32.1)	0.05
Thiazolidinediones	609 (9.8)	198 (5.7)	807 (8.3)	0.15
Other	477 (7.7)	260 (7.5)	737 (7.6)	0.01
Blood pressure, mean (SD)				
Systolic	130.1 (17.0)	131.3 (17.5)	130.5 (17.2)	0.07
Diastolic	73.8 (10.9)	73.5 (11.6)	73.7 (11.2)	0.02
Blood pressure category				
Normal	1473 (23.9)	779 (22.6)	2252 (23.4)	0.06
Prehypertensive	2991 (48.5)	1626 (47.1)	4617 (48.0)	
Stage 1	1320 (21.4)	812 (23.5)	2132 (22.2)	
≥Stage 2	379 (6.2)	236 (6.8)	615 (6.4)	
Missing	70 (1.1)	24 (0.7)	94 (1.0)	
Charlson-Elixhauser category, mean (SD)	−0.082 (0.97)	−0.103 (1.02)	−0.089 (0.99)	0.02
Health conditions				
Anxiety	1274 (20.4)	734 (21.1)	2008 (20.7)	0.02
Depression	2157 (34.6)	1053 (30.3)	3210 (33.1)	0.09
Diabetes	5952 (95.5)	3221 (92.6)	9173 (94.5)	0.12
Deep-vein thrombosis	38 (0.6)	28 (0.8)	66 (0.7)	0.02
Dyslipidemia	4775 (76.6)	2659 (76.5)	7434 (76.6)	0.00
Eating disorder	969 (15.6)	231 (6.6)	1200 (12.4)	0.29
Gastroesophageal reflux disease	2609 (41.9)	1264 (36.4)	3873 (39.9)	0.11
Hypertension	5113 (82.0)	2729 (78.5)	7842 (80.8)	0.09
Infertility	29 (0.5)	29 (0.8)	58 (0.6)	0.05
Kidney disease	1268 (20.3)	670 (19.3)	1938 (20.0)	0.03
Nonalcoholic fatty liver disease	1914 (30.7)	730 (21.0)	2644 (27.2)	0.22
Osteoarthritis	148 (2.4)	93 (2.7)	241 (2.5)	0.02
Polycystic ovarian syndrome	257 (4.1)	147 (4.2)	404 (4.2)	0.01
Pulmonary embolism	87 (1.4)	39 (1.1)	126 (1.3)	0.03
Psychotic disorder	197 (3.2)	96 (2.8)	293 (3.0)	0.02
Sleep apnea	3607 (57.9)	1740 (50.0)	5347 (55.1)	0.16
Smoker	582 (9.3)	276 (7.9)	858 (8.8)	0.05
Substance use disorder	143 (2.3)	102 (2.9)	245 (2.5)	0.04
Inpatient hospital days in y before surgery, mean (SD)	0.67 (8.0)	0.83 (8.0)	0.73 (8.0)	0.02
Inpatient hospital days in categories				
0	5758 (92.4)	3156 (90.8)	8914 (91.8)	0.06
1-7	373 (6.0)	253 (7.3)	626 (6.5)	
8-14	45 (0.7)	36 (1.0)	89 (0.9)	
15 or more	57 (0.9)	32 (0.9)	81 (0.8)	
DiaRem score^a^				
0-2	809 (13.0)	517 (14.9)	1326 (13.7)	0.11
3-7	2211 (35.5)	1251 (36.0)	3462 (35.7)	
8-12	759 (12.2)	412 (11.9)	1171 (12.1)	
13-17	2127 (34.1)	1185 (34.1)	3312 (34.1)	
18-22	327 (5.3)	112 (3.2)	439 (4.5)	
Missing	0 0	0 0	0 0	

Abbreviations: BMI, body mass index (calculated as weight in kilograms divided by height in meters squared); *GLP-1*, glucagon-like peptide 1; IQR, interquartile range; NA, not applicable.

^a^ Score indicates preoperative prognostication of type 2 diabetes remission following Roux-en-Y gastric bypass surgery, where a higher score indicates lower probability of type 2 diabetes remission: 0 to 2 (88%-99%), 3 to 7 (64%-88%), 8 to 12 (23%-49%), 13 to 17 (11%-33%), and 18 to 22 (2%-16%).

Logistic regression models estimating treatment propensity scores included fixed main effects for the prespecified covariates plus baseline variables for automated selection. To allow for differing outcomes of confounding variables by procedure site, propensity score models included subsets of all possible 2-way interactions between the listed variables and site. The subset of interactions and the additional covariates beyond the prespecified set were chosen using the least absolute shrinkage and selection operator method, with cross validation to select the most parsimonious model, with prediction error close to the minimum possible (within 1 SE).^32^

Follow-up for T2DM remission was calculated from the index procedure date to the last observable data point following surgery (ie, the last observed visit, weight, blood pressure, HbA_1c_ laboratory value, or diabetes prescription). Remission analyses’ censoring events included death, conversion to a second bariatric procedure (eg, SG to RYGB), pregnancy (at the delivery date minus 270 days), and an 18-month lapse in diabetes-specific health care at participating sites. The relapse analyses included an additional censoring event, lapse in provision of any care, because patients in remission from T2DM were not necessarily expected to receive HbA_1c_ measures or T2DM prescriptions but needed to receive care in the system to be observed for relapse. It was defined as more than 18 months without any recorded HbA_1c_ levels, body weight measurement, blood pressure, diagnosis code, or procedure code. Since inpatient hospitalization can temporarily worsen glycemic control, we excluded HbA_1c_ measurements from admission date to 90 days after discharge and medication orders from admission dates to the day before discharge.

#### Subgroup Analyses

Exploratory hypothesis-generating analyses examined heterogeneity of treatment outcomes. Following recommendations for use of risk-stratified analyses to detect differences in treatment outcome,^33^ subgroups defined by DiaRem score (Table 1) were assessed via interactions with procedure type. The DiaRem score is a widely validated approach to preoperative prognostication of T2DM remission after bariatric surgery; higher scores denote a lower probability of T2DM remission.^34^ It is calculated based on age, HbA_1c_ level, insulin use, and use of oral diabetes medications.

#### Secondary Analyses

Estimates of trends in mean total weight loss percentage were obtained using linear mixed-effects modeling with weight as the outcome and potential confounders (including baseline weight) and deciles of the propensity score as the independent variables. Adjusted total weight loss percentage was computed as the percentage change between the mean weight and the mean baseline weight. Time to T2DM relapse was assessed among patients who experienced diabetes remission, using the same methods as in the remission analyses. Adjusted absolute changes in HbA_1c_ level at 1 year, 3 years, and 5 years following surgery were estimated by procedure using a linear mixed-modeling framework with random effects for individual (intercept) and follow-up time (slope). A b-spline basis included a smooth function of follow-up time in the model, allowing nonlinearity in the trajectory of percentage change in HbA_1c_ level following surgery. For HbA_1c_ level, we considered less than 7% as a goal range, consistent with American Diabetes Association goals for adults who are not pregnant, and more than 8% (well above the goal for many adults, including those with advanced vascular complications) to indicate poor control.^35^

#### Sensitivity Analyses

Sensitivity analyses considered 9-month and 12-month alternative lags from the last observed T2DM medication order to define remission (HbA_1c_ level <6.5%). To evaluate variability in medication data capture across different health systems, the primary analyses were repeated using only data from 8 integrated health systems, where infrastructure may enable more complete access to medication orders. Additional sensitivity analyses assessed 2 alternate censoring scenarios for inpatient stays: (1) no removal of inpatient medications or HbA_1c_ values and (2) censoring follow-up at the day of admission. Similar sensitivity analyses were applied to the relapse analyses. Analyses were conducted using R version 3.4.3 (R Foundation for Statistical Computing).

## Results

### Sample

In this unmatched surgical cohort, the analytic sample included 9710 adults, primarily female (7051 female patients [72.6%]) with a mean (SD) age of 49.8 (10.5) years (Table 1). A total of 6233 (64.2%) underwent RYGB, and 3477 (35.8%) had SGs. The mean (SD) preoperative BMI was 49.0 (8.4). Patients were primarily white (6040 [72.2%]). Most (7904 [81.4%]) surgeries occurred between 2010 and 2014.

The mean (SD) preoperative HbA1c was 7.2% (1.3%), and patients took a mean (SD) of 1.66 (1.1) diabetes medications (range, 0-7 medications). The mean (SD) preoperative systolic and diastolic blood pressure were 130.5 (17.2) mm Hg and 73.7 (11.2) mm Hg, respectively. Weight-associated comorbidities were common. Patients who had RYGB had higher prevalence of some comorbidities, such as sleep apnea (RYGB: 3607 patients [57.9%]; SG: 1740 patients [50.0%]), nonalcoholic fatty liver disease (RYGB: 1914 patients [30.7%]; SG: 730 patients [21.0%]), and gastroesophageal reflux disease (RYGB: 2609 patients [41.9%]; SG: 1264 patients [36.4%]). The mean (SD) Charlson/Elixhauser score was negative (−0.089 [0.99]), consistent with the high hypertension prevalence in an otherwise relatively healthy sample.

### Percentage of Total Weight Lost

**Figure 1.**
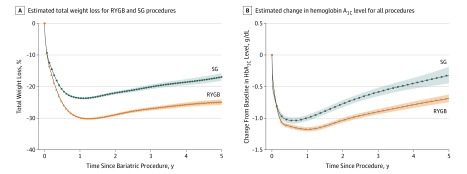
Adjusted Total Weight Loss and Change in Hemoglobin A_1c_ Level by Procedure Over 5 Years of Follow-up Shaded areas represent 95% pointwise CIs for procedure-specific changes in hemoglobin A_1c_ levels. RYGB indicates Roux-en-Y gastric bypass; SG, sleeve gastrectomy.

Patients who had each procedure showed considerable weight loss 1 year after surgery (SG, −22.8% [95% CI, −23.1% to −22.5%]; RYGB, −29.1% [95% CI, −29.3% to −28.8%]); typically, weight regain then occurred. The groups maintained a mean body weight well below the baseline at 5 years (SG, −16.1% [95% CI, −17.3% to −14.8%]; RYGB, −24.1% [95% CI, −25.0% to −23.3%]). Typically, the RYGB group reflected 6.2% to 8.1% more total body weight loss than the SG group at each point (Figure 1; Table 2). This represents a 10.2-kg difference (95% CI, 8.3-12.1 kg; *P* < .001) in weight loss between RYGB and SG at 5 years.

**Table 2.** Comparative Effectiveness of Gastric Bypass and Sleeve Gastrectomy for Percentage of Total Weight Loss and Absolute Difference in Hemoglobin A_1c_ Level Among Adults With Diabetes With 1, 3, and 5 Years of Follow-up^a^

Group	Time Since Bariatric Procedure					
	1 y		3 y		5 y	
	Patients, No.	Finding	Patients, No.	Finding	Patients, No.	Finding
Total weight loss, %						
Sleeve gastrectomy	2404	−22.8 (−23.1 to −22.5)	2404	−19.2 (−20.0 to −18.5)	2404	−16.1 (−17.3 to −14.8)
Roux-en-Y gastric bypass	4025	−29.1 (−29.3 to −28.8)	4025	−26.2 (−26.7 to −25.7)	4025	−24.1 (−25.0 to −23.3)
Difference	NA	6.2 (5.8-6.7)	NA	7.0 (6.1-7.9)	NA	8.1 (6.6-9.6)
*P* Value	NA	<.001	NA	<.001	NA	<.001
Hemoglobin A_1c_ mean difference (95% CI), %^a^						
Sleeve gastrectomy	2935	−0.89 (−0.93 to −0.86)	2935	−0.56 (−0.64 to −0.49)	2935	−0.35 (−0.51 to −0.19)
Roux-en-Y gastric bypass	5428	−1.12 (−1.14 to −1.09)	5428	−1.01 (−1.06 to −0.97)	5428	−0.80 (−0.88 to −0.72)
Difference	NA	−0.22 (−0.26 to −0.18)	NA	−0.45 (−0.54 to −0.36)	NA	−0.45 (−0.63 to −0.27)
*P* Value	NA	<.001	NA	<.001	NA	<.001

Abbreviations: *ICD-9-CM*, *International Classification of Diseases, Ninth Revision, Clinical Modification*; NA, not applicable; SNOMED, Systematized Nomenclature of Medicine.

^a^ Difference is the baseline value minus the end point value; the model was adjusted for age, sex, race, Hispanic ethnicity, body mass index (calculated as weight in kilograms divided by height in meters squared), hemoglobin A_1c_ value, blood pressure, number of inpatient hospital days in the year prior to surgery, number of diabetes medications excluding insulin, insulin use, Charlson/Elixhauser comorbidity score, year of procedure, days from hemoglobin A_1c_ measurement to baseline, having an *ICD-9-CM* or SNOMED code for diabetes, smoking, having an *ICD-9-CM* or SNOMED code for other comorbidities (hypertension, dyslipidemia, sleep apnea, osteoarthritis, nonalcoholic fatty liver disease, gastroesophageal reflux disease, depression, anxiety, eating disorder, substance use, psychosis, kidney disease, infertility, polycystic ovarian syndrome, deep-vein thrombosis, and pulmonary embolism), having *ICD-9-CM* or SNOMED codes for specific diabetes medications (biguanides, glucagon-like peptide–1 agonists, sulfonylureas, thiazolidinediones, and others), site, and propensity-score deciles.

### T2DM Remission

**Figure 2.**
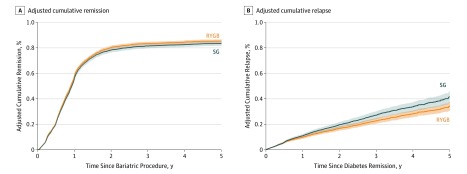
Cumulative Incidence Rates of Type 2 Diabetes Remission and Relapse Across 5 Years in the National Patient-Centered Clinical Research Network Bariatric Study Cohort Shaded areas represent 95% pointwise CIs for procedure-specific rates. RYGB indicates Roux-en-Y gastric bypass; SG, sleeve gastrectomy.

The cohort was followed up for a median of 2.7 (interquartile range, 1.26-4.19) years. Type 2 diabetes remission occurred primarily in the first 2 years (Figure 2). Patients who underwent RYGB showed slightly (10%) higher T2DM remission rates than those who had SG (hazard ratio, 1.10 [95% CI, 1.04-1.16]; Table 3). We estimated that 59.2% (95% CI, 57.7%-60.7%) of patients who had RYGB vs 55.9% (95% CI, 53.9%-57.9%) of those who had SG experienced remission by 1 year, 84.3% (95% CI, 82.9%-85.5%) vs 81.5% (95% CI, 79.6%-83.2%) at 3 years, and 86.1% (95% CI, 84.7%-87.3%) vs 83.5% (95% CI, 81.6%-85.1%) at 5 years (Table 3).

**Table 3.** Adjusted Hazard Ratios Comparing Time to Remission Since Surgery With Time to Relapse Since Remission for Roux-en-Y Gastric Bypass vs Sleeve Gastrectomy

Outcome	Total Patients, No.	Time Since Bariatric Procedure									Adjusted Hazard Ratio (95% CI)	*P* Value
		1 y			3 y			5 y				
		No. at Risk^a^	Cumulative Events^b^	Estimated Cumulative % (95% CI)	No. at Risk	Cumulative Events	Estimated Cumulative % (95% CI)	No. at Risk	Cumulative Events	Estimated Cumulative % (95% CI)		
Type 2 diabetes remission												
Roux-en-Y gastric bypass	5428	1800	2825	59.2 (57.7-60.7)	557	3593	84.3 (82.9-85.5)	215	3620	86.1 (84.7-87.3)	1.10 (1.04-1.16)^c^	.007
Sleeve gastrectomy	2935	917	1519	55.9 (53.9-57.9)	211	1880	81.5 (79.6-83.2)	27	1889	83.5 (81.6-85.1)	1 [Reference]	
Type 2 diabetes relapse^d^												
Roux-en-Y gastric bypass	3352	2273	367	8.4 (7.4-9.3)	1053	665	21.2 (19.1-23.2)	264	772	33.1 (29.6-36.5)	0.75 (0.67-0.84)^d^	<.001
Sleeve gastrectomy	1751	917	199	11.0 (9.6-12.4)	211	369	27.2 (24.1-30.1)	27	400	41.6 (36.8-46.1)	1 [Reference]	

Abbreviations: *ICD-9-CM*, *International Classification of Diseases, Ninth Revision, Clinical Modification*; NA, not applicable; SNOMED, Systematized Nomenclature of Medicine.

^a^ Number of people still being followed up at each point.

^b^ Number of people who had an event in the relevant time frame.

^c^ For Roux-en-Y gastric bypass vs sleeve gastrectomy; remission of diabetes was defined as hemoglobin A_1c_ less than 6.5% after 6 months without any prescription order for a diabetes medication; covariates included age, sex, race, Hispanic ethnicity, body mass index (calculated as weight in kilograms divided by height in meters squared), hemoglobin A_1c_, blood pressure, days from body mass index measurement to baseline, number of inpatient hospital days in the year prior to surgery, number of diabetes medications excluding insulin, insulin use, Charlson/Elixhauser comorbidity score, year of procedure, having an *ICD-9-CM* or SNOMED code for diabetes, smoking, having an *ICD-9-CM* or SNOMED code for other comorbidities (hypertension, dyslipidemia, sleep apnea, osteoarthritis, nonalcoholic fatty liver disease, gastroesophageal reflux disease, depression, anxiety, eating disorder, substance use, psychosis, kidney disease, infertility, polycystic ovary syndrome, deep-vein thrombosis, or pulmonary embolism), having *ICD-9-CM* or SNOMED codes for specific diabetes medications (biguanides, glucagon-like peptide–1 agonists, sulfonylureas, thiazolidinediones, and others), site, and propensity-score deciles.

^d^ Relapse of diabetes was defined as occurrence of any hemoglobin A_1_c level of 6.5% or more and/or prescription order for a diabetes medication. Covariates included age, sex, race, Hispanic ethnicity, body mass index, hemoglobin A_1c_ level, blood pressure, days from body mass index measurement to baseline, a number of inpatient hospital days in the year prior to surgery, a number of diabetes medications excluding insulin, insulin use, Charlson/Elixhauser comorbidity score, the year of procedure, having an *ICD-9-CM* or SNOMED code for diabetes, smoking, having an *ICD-9-CM* or SNOMED code for other comorbidities (hypertension, dyslipidemia, sleep apnea, osteoarthritis, nonalcoholic fatty liver disease, gastroesophageal reflux disease, depression, anxiety, eating disorder, substance use, psychosis, kidney disease, infertility, polycystic ovarian syndrome, deep vein thrombosis, or pulmonary embolism), having *ICD-9-CM* or SNOMED codes for specific diabetes medications (biguanides, GLP-1 agonists, sulfonylureas, thiazolidinediones, and others), site, and propensity-score deciles.

Sensitivity analyses requiring 9-month and 12-month time frames without a diabetes medication prescription to define remission produced similar results to the primary analysis and its 6-month time frame, although differences between SG and RGB were not always statistically significant (eTable 2 in the Supplement). Analyses restricted to 8 integrated health systems yielded qualitatively similar results to the primary analyses, despite slightly higher cumulative remission rates for SG and RYGB (eTable 3 in the Supplement).

### T2DM Relapse

A total of 6141 patients with documented T2DM remission were eligible for the relapse analyses. Preoperation demographic and health features were similar to those of the larger T2DM cohort (eTable 4 in the Supplement). Mean (SD) preoperation HbA_1c_ levels were slightly lower (7.0% [1.1%]) vs 7.2% [1.3%]) as was the mean (SD) number of diabetes medications (1.5 (1.1) medications vs 1.7 [1.1] medications) and insulin use (2317 of 6141 [37.7%] vs 4692 of 9710 [48.3%]; eTable 4 in the Supplement). They were followed up for relapse for a median of 2.4 (0.003-10.35) years.

The T2DM relapse rate was lower for RYGB than SG (hazard ratio, 0.75 [95% CI, 0.67-0.84]). Estimated proportions of relapse for the RYGB and SG groups, respectively, were 8.4% (95% CI, 7.4%-9.3%) and 11.0% (95% CI, 9.6%-12.4%) 1 year after remission, 21.2% (95% CI, 19.1%-23.2%) and 27.2% (95% CI, 24.1%-30.1%) at 3 years, and 33.1% (95% CI, 29.6%-36.5%) and 41.6% (95% CI, 36.8%-46.1%) at 5 years (Table 3). Sensitivity analyses showed similar findings (eTable 5 and eTable 6 in the Supplement).

### Change in Glycosylated Hemoglobin

Patients who underwent RYGB experienced larger and more-sustained HbA_1c_ reductions than those using SG (Figure 1). In adjusted comparisons, patients who had RYGB showed a 1.12 percentage point drop in HbA_1c_ level (95% CI, 1.09-1.14 percentage points) over 1 year. This change was 0.22 (95% CI, 0.18-0.26) percentage points lower than seen for patients who had SG (Table 2). At 5 years, HbA_1c_ levels remained 0.80 (95% CI, 0.72-0.88) percentage points below baseline among patients who had RYGB and 0.35 (95% CI, 0.19-0.51) percentage points below baseline among patients who had SG, a difference of 0.45 (95% CI, 0.27-0.62) percentage points. The proportion with a poorly controlled HbA_1c_ level (≥8.0%) declined from baseline through 1 year of follow-up for both groups (patients who had RYGB, 24.6% [95% CI, 23.5%-25.7%] to 6.7% [95% CI, 6.0%-7.7%]; patients who had SG, 17.5% [95% CI, 16.24%-18.88%] to 8.3% [95% CI, 7.05%-9.79%]); it then increased, with 16.2% of patients who had RYGB and 22.4% of patients who had SG having HbA_1c_ levels greater than 8.0% 5 years after surgery. Following surgery, a well-controlled HbA_1c_ level (<6.5%) was consistently more common among patients who had RYGB (eFigure 2 in the Supplement).

### T2DM Remission in Patient Subgroups

Analyses for heterogeneity of treatment outcomes indicated that the likelihood of diabetes remission comparing RYGB vs SG varied significantly across DiaRem strata (eTable 7 in the Supplement). Patients with higher DiaRem scores showed greater likelihood of diabetes remission with RYGB compared with SG, with a statistically significant association for scores between 13 and 17. Among individuals with DiaRem scores in the 13-point to 17-point range, 83.4% (95% CI, 77.9%-87.6%) of patients who had RYGB had experienced T2DM remission by 5 years of follow-up vs 76.6% (95% CI, 70.0%-81.8%) of patients who had SG (eTable 8 in the Supplement).

## Discussion

In this sample of US adults with T2DM and bariatric surgery, 56% to 59% of those with RYGB or SG experienced T2DM remission in the year following surgery and 84% to 86% did so within 5 years of follow-up. However, T2DM relapse was common; 33% of patients who had RYGB and 42% of patients who had SG relapsed within 5 years of initial remission. The glycemic control of patients who had RYGB and SG showed sustained improvements from the samples’ baseline mean HbA_1c_ level of 7.2%, with an estimated mean HbA_1c_ level 0.80 percentage points below baseline for the RYGB group 5 years after surgery vs 0.35 percentage points below baseline for the SG group. While both groups experienced considerable weight loss, patients who had RYGB lost more weight and maintained weight loss better than did patients who had SG.

Overall, these results indicate that RYGB is associated with better long-term T2DM and weight outcomes than SG in real-world clinical settings. This is at odds with recent randomized clinical trials that compared T2DM outcomes of RYGB and SG and found no significant differences.^19,20,21^ Those trials had longer duration of follow-up but much smaller sample sizes, which may have limited their power to detect differences between the procedures. Also, patients who are willing to undergo randomization between RYGB and SG and surgeons who have equal skill and equipoise for RYGB and SG are likely different from those who choose either RYGB or SG in uncontrolled settings. Thus, while the more rigorous, randomized clinical trial data indicate that RYGB and SG perform similarly in highly controlled environments, in everyday practice, the outcome differences may be larger.

As expected,^1,6,7,22,36^ some patient subgroups showed lower rates of T2DM remission. Our findings indicate that patients with lower preoperative probability for T2DM remission (11%-33%) may be more likely to achieve T2DM remission with RYGB compared with SG. Estimating the likelihood of T2DM remission could help inform patients’ and clinicians’ discussions of procedure choice. Preoperative insulin use, older age, higher HbA_1c_ level, and more complex T2DM medication regimens predispose patients to lower probability of T2DM remission in the DiaRem scoring system.^34^ Informed decision-making for procedure choice should also consider other factors, such as the potential for adverse events.

A range of T2DM remission rates are found in studies of bariatric surgery,^6,7,12,37,38,39,40,41^ reflecting varying follow-up time, remission definitions, and population characteristics (eg, insulin use, HbA_1c_ level).^38^ The cumulative remission rates over 80% for SG or RYGB in PBS are consistent with or somewhat higher than estimates from systematic reviews or meta-analyses (54%-78%)^4,37,40^ and similar to findings (72%; all procedures) from 3 US health systems.^6^ Literature on T2DM relapse is more limited. Published relapse estimates range from approximately 25% to 53%^7,12,41^ and are typically calculated across a mix of procedure types and time frames; those ranges are consistent with PBS’s 5-year cumulative relapse rates.

The large PBS sample and its comparison of remission and relapse rates across procedures, extended follow-up, and evaluation of remission across patient subgroups contribute unique insight to the literature. Findings also contribute to ongoing dialogue about leveraging real-world evidence to understand health and improve care.^42,43,44^ Such data can reflect generalizable populations of patients and clinicians, as well as actual health care practices and settings.^44^ The data standardization and curation processes of PCORnet^45^ help mitigate data quality concerns that have been raised regarding analyses of electronic health record data,^42,44^ as do the consistency of our findings with prior literature. Our analyses suggest that, coupled with rigorous attention to study design and analytic methods, PCORnet data can be a valuable resource for health research.

### Limitations

This study has limitations. Because of the observational study design, procedure choice may have been influenced by unmeasured factors that impact the surgical effect on diabetes. Despite direct adjustment and the use of propensity scores, confounding may persist. Using *ICD-9-CM* codes to assess baseline health may underestimate comorbidity prevalence. The PBS definitions for T2DM relapse and remission rely on medication-prescribing data. To the extent that prescriptions were not filled, medication use may be overestimated. Some patients may have had T2DM medications ordered outside of the health systems in the study. All dates were normalized to the date of surgery, so within a calendar year, we cannot differentiate patients with loss to follow-up from those for whom the study end date had been reached. Future work should address the potential role of weight loss in mediating diabetes remission and relapse.

Similar to prior research,^7^ 19% of the cohort was not prescribed diabetes medication preoperatively. Some people may have used lifestyle alone to treat diabetes.^46^ Undiagnosed diabetes is common,^47^ and others may have been diagnosed during the preoperative evaluation—emphasizing the importance of care coordination between medical and surgical health professions among patients considering bariatric surgery.

## Conclusions

In conclusion, among patients with T2DM who underwent RYGB or SG, most experienced T2DM remission at some point over 5 years of follow-up. While SG and RYGB resulted in similar rates of initial T2DM remission, RYGB was associated with larger and more persistent improvements in glycemic control and 25% lower rates of T2DM relapse compared with SG. Patients with more advanced T2DM at the time of surgery for whom remission is more difficult to achieve (eg, those with older age, insulin use, more complex T2DM medications, and/or poor glycemic control) may expect larger improvements in T2DM with RYGB compared with SG. On the other hand, for patients with higher likelihood of T2DM remission, RYGB and SG are likely to yield similar 5-year T2DM outcomes. For patients, clinicians and policy makers to make informed decisions about which procedure is best suited to patients’ personal situations, additional data are needed to understand the adverse event profile of the procedures as well as patient values regarding procedure choice and the role of surgery relative to other aspects of lifelong weight management.

## Notes

Supplement. **eTable 1.** List of Clinical Data Research Networks and Sites participating in the PCORnet Bariatric Study **eFigure 1.** Flow diagram for cohort selection **eAppendix.** Eligibility criteria for PBS Diabetes Analyses **eTable 2.** Remission findings using 9- and 12-month censoring options **eTable 3.** Remission findings with sample restricted to IHPs **eTable 4.** Descriptive features of patients examined in the relapse analysis **eTable 5.** Relapse findings using different approaches to censoring **eTable 6.** Relapse findings restricted to Integrated Health Plans **eFigure 2.** Unadjusted proportion of patients with well-controlled (<6.5%; left panel) or poorly controlled (≥8%) HbA1c **eTable 7.** Comparison of Type 2 Diabetes Remission Rates at 1, 3, and 5 Years across Subgroups **eTable 8.** Estimated percentage of T2DM remission by type of procedure and DiaRem Score **eAppendix 2. **Comparing Adjustable Gastric Banding to Roux-en-Y Gastric Bypass and Sleeve Gastrectomy
