# Supplementary material for: Comparing the 5-Year Diabetes Outcomes of Sleeve Gastrectomy and Gastric Bypass: The National Patient-Centered Clinical Research Network (PCORNet) Bariatric Study
Source: JAMA Surg. 2020 Mar 4;155(5):e200087. doi: 10.1001/jamasurg.2020.0087 (PMC7057171; doi:10.1001/jamasurg.2020.0087)

Supplementary Online Content

McTigue KM, Wellman RD, Nauman E, et al; for the PCORnet Bariatric Study Collaborative. Comparing the 5-year diabetes outcomes of sleeve gastrectomy and gastric bypass. *JAMA Surg*. Published online March 4, 2020. doi:10.1001/jamasurg.2020.0087

eTable 1. List of Clinical Data Research Networks and Sites participating in the PCORnet Bariatric Study

eFigure 1. Flow diagram for cohort selection

eAppendix. Eligibility criteria for PBS Diabetes Analyses

eTable 2. Remission findings using 9- and 12-month censoring options

eTable 3. Remission findings with sample restricted to IHPs

eTable 4. Descriptive features of patients examined in the relapse analysis

eTable 5. Relapse findings using different approaches to censoring

eTable 6. Relapse findings restricted to Integrated Health Plans

eFigure 2. Unadjusted proportion of patients with well-controlled (<6.5%; left panel) or poorly controlled (≥8%) HbA1c

eTable 7. Comparison of Type 2 Diabetes Remission Rates at 1, 3, and 5 Years across Subgroups

eTable 8. Estimated percentage of T2DM remission by type of procedure and DiaRem Score

This supplementary material has been provided by the authors to give readers additional information about their work.

© 2020 McTigue KM et al. *JAMA Surgery.*

**eTable 1: List of CDRNs & Sites participating in the PCORnet Bariatric Study**	
**Clinical Data Research Networks (CDRNs)**	**Data-contributing Sites**
Chicago Area Patient-Centered Outcomes Research Network (CAPriCORN)	Loyola Medicine
	Northwestern Medicine
	University of Chicago Medical Center
	University of Illinois Hospital & Health Science System
Greater Plains Collaborative (GPC)	Marshfield Clinic
	University of Texas Southwestern Medical Center
	University of Iowa Healthcare[a]
	University of Kansas Medical Center
	University of Wisconsin – Madison[a]
	University of Nebraska Medical Center[a]
Kaiser Permanente & Strategic Partners Patient Outcomes Research To Advance Learning (PORTAL)	Kaiser Permanente Washington Health Research Institute (formerly Group Health Research Institute)
	HealthPartners Research Foundation
	Kaiser Permanente Colorado
	Kaiser Permanente Mid-Atlantic
	Kaiser Permanente Northwest
	Kaiser Permanente Southern California
Mid-South	Greenway[a]
	University of North Carolina
	Vanderbilt University Medical Center
New York City Clinical Data Research Network (NYC-CDRN)	Mount Sinai[a]
	New York University[a]
	Weill Cornell
	Montefiore/Einstein
OneFlorida Clinical Research Consortium	University of Florida Health
	Orlando Health
	Tallahassee Memorial Health System
PaTH Towards a Learning Health System Clinical Data Research Network (PaTH)	Geisinger Health System
	Johns Hopkins University and Health Plan
	Penn State College of Medicine, Penn State Milton S. Hershey Medical Center
	Temple Health System, Lewis Katz School of Medicine at Temple University
	University of Pittsburgh and University of Pittsburgh Medical Center (UPMC)
	UPMC Health Plan[a]
	University of Utah and University of Utah Health Care
A Pediatric Learning Health System (PEDSnet)	Cincinnati Children's Hospital Medical Center
	Nemours[a]
	Nationwide Children's Hospital[a]
Patient-Centered SCAlable National Network for Effectiveness Research (pSCANNER)	University of California Irvine[a]
	University of California Los Angeles
Research Action for Health Network (REACHnet)	Baylor Scott & White Health
	Ochsner Health System
	Tulane University[a]
Scalable Collaborative Infrastructure for a Learning Healthcare System (SCILHS)	Beth Israel Deaconess Medical Center
	Boston HealthNet
	Partners Health
	Wake Forest Baptist Hospital

*Did not contribute data to the current analyses of T2DM outcomes

© 2020 McTigue KM et al. *JAMA Surgery.*

eFigure 1: Cohort Selection

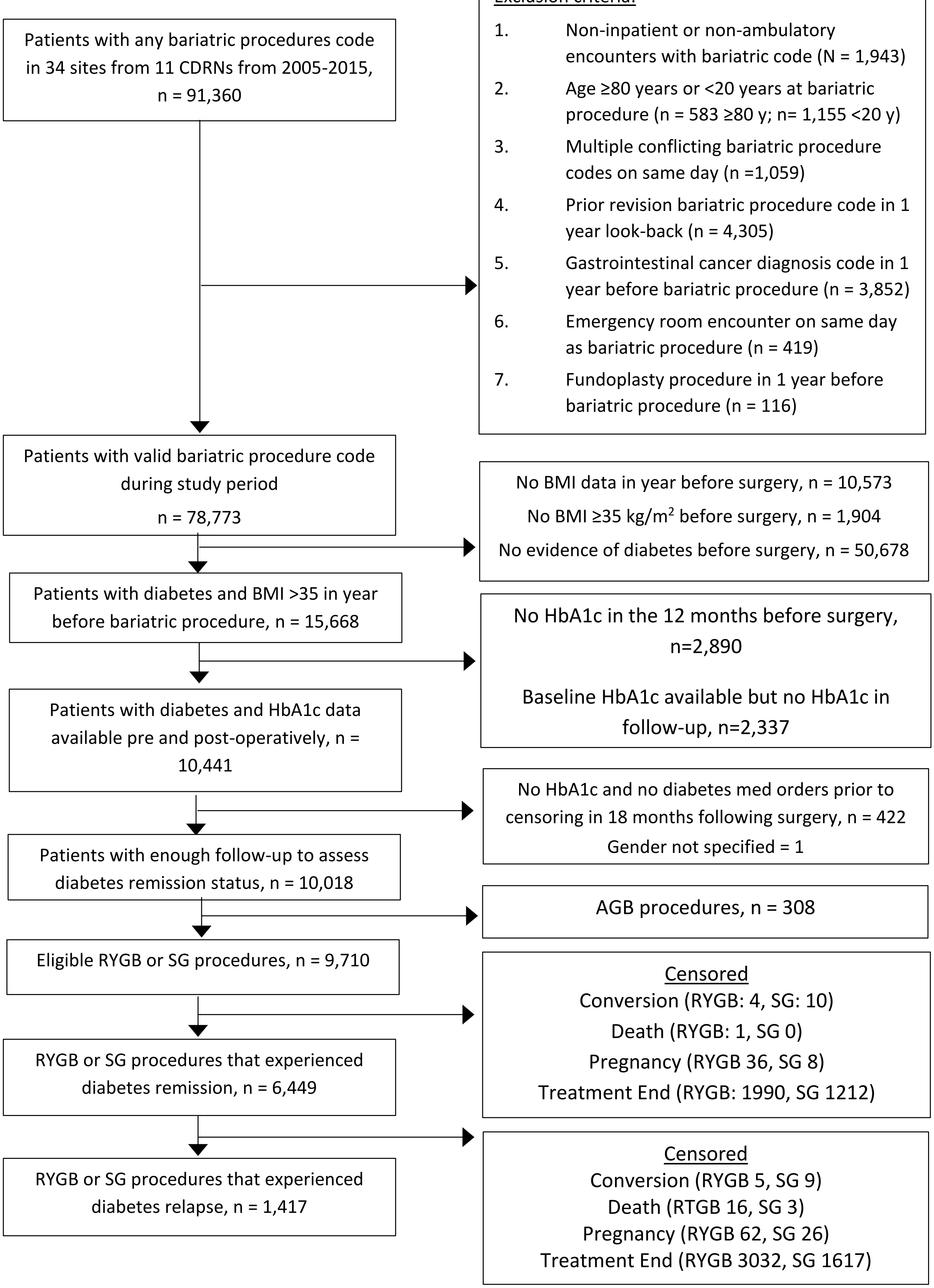

© 2020 McTigue KM et al. *JAMA Surgery.*

eAppendix. Eligibility criteria for PBS Diabetes Analyses

Exclusions made to the group of 91,360 patients who were identified as having undergone bariatric surgery during the time-frame of interest. We excluded: 1) patients with non-inpatient or non-ambulatory encounters that had a bariatric code (n = 1,943); 2) age ≥80 (n=583) or <20 (n=1,155) at the time of the bariatric procedure; 3) individuals with multiple conflicting bariatric procedure codes on same day (n =1,059); 3) any revision bariatric procedure code (n = 4,305), gastrointestinal cancer diagnosis code (n=3,852), or fundoplasty procedure (n = 116) in the year before the index procedure; 4) any emergency room encounter on the day of index procedure (n = 419); and 5) patients without a body mass index (BMI; n = 10,573) and those without a BMI ≥35 kg/m^2 (n = 1,904) in the year before their procedure. This left us with 66,296 adults with an eligible bariatric procedure. We then removed all patients without evidence of DM in the year prior to their bariatric procedure (n = 50,628), where evidence of DM was defined as 1) a HbA1c ≥6.5% at the most recent measurement prior to surgery or 2) prescription of any oral or injectable DM medication in the year prior to surgery. Patients taking only metformin, thiazolidinedione, or liraglutide in the year prior to surgery also needed an ICD-9 or SNOMED code for DM or a HbA1c ≥6.5% in the year prior to surgery to be considered as having DM at baseline. We excluded patients with evidence of Type 1 diabetes, which was defined as having both 1) use of insulin alone, and 2) >50% of DM diagnosis codes indicating Type 1 DM in the year before surgery. This left us with 15,668 patients with presumed type 2 DM in the year prior to an eligible bariatric procedure (top box, Figure 1).

Next, we excluded patients with insufficient data for analysis of our DM outcomes (eFigure 1), including: 1) patients with no HbA1c in the 12 months prior to surgery (n = 2,890), 2) those with no HbA1c recorded after surgery (n = 2,337), and 3) patients with insufficient follow-up for DM-related care defined as no HbA1c measure or DM medication prescription order within the first 18 months after surgery (n = 422). Finally, we excluded 308 patients who had an adjustable gastric banding procedure.

Our final analytic sample included 9,710 patients who met all eligibility criteria and had undergone either a RYGB or SG procedure.

© 2020 McTigue KM et al. *JAMA Surgery.*

eTable 2: Adjusted hazard ratios comparing time to remission using a 9-month (top panel) or 12-month (bottom panel) cut point for censoring due to remission*

	Adjusted HR* (95% CI)	P-Value	Estimated cumulative percentage of patients with T2DM who had experienced remission (95% CI) at different times of follow-up			
				1 year	3 years	5 years
9 months	1.00 (0.94, 1.06)	0.989	RYGB	40.2 (38.7, 41.6)	76.9 (75.3, 78.4)	79.3 (77.7, 80.9)
			SG	40.2 (38.3, 42.0)	76.9 (74.8, 78.9)	79.4 (77.2, 81.3)
12 months	1.1 (1.0, 1.2)	0.042	RYGB	18.1 (17, 19.1)	67.1 (65.1, 69.0)	70.4 (68.3, 72.3)
			SG	16.8 (15.6, 18)	64.2 (61.6, 66.6)	67.5 (64.8, 69.9)

© 2020 McTigue KM et al. *JAMA Surgery.*

eTable 3: Adjusted hazard ratios comparing time to remission for RYGB versus SG, with sample restricted to integrated health system[a]

Comparison	Adjusted HR* (95% CI)	P-Value	Estimated cumulative percentage of patients with T2DM who had experienced remission (95% CI) at different times of follow-up			
				1 year	3 years	5 years
RYGB vs SG	1.10 (1.04, 1.18)	0.009	RYGB	62.8 (61.0, 64.4)	85.7 (84.3, 87.0)	87.4 (85.9, 88.6)
			SG	59.1 (56.7, 61.4)	82.8 (80.7, 84.7)	84.6 (82.6, 86.5)

[a]Integrated health systems include those systems that integrate health care delivery with health insurance coverage: Kaiser Permanente (Southern California, Northwest, Colorado, Mid-Atlantic States, and Washington), Health Partners, Geisinger Health System, and Marshfield Clinic

© 2020 McTigue KM et al. *JAMA Surgery.*

eTable 4: Descriptive features of patients examined in the relapse analysis; the sample is described prior to bariatric surgery.

	RYGB		SG		Overall		Std. Diff.
N (row %)	4039	(65.8)	2102	(34.2)	6141	(100.0)	
Follow-up time (years)							
Mean (SD)	2.9	(2.0)	1.9	(1.3)	2.5	(1.9)	
Median (IQR)	2.8	(2.8)	1.7	(2.2)	2.4	(2.7)	
Minimum	0.0027		0.0027		0.0027		
Maximum	10.4		6.7		10.4		
Female, N (%)	3011	(74.6)	1517	(72.2)	4528	(73.8)	0.05
Age, mean (SD)	49.5	(10.4)	49.2	(10.8)	49.4	(10.5)	0.03
Age category, N (%)							
20-44 years	1311	(32.5)	725	(34.5)	2036	(33.2)	0.05
45-64 years	2430	(60.2)	1218	(57.9)	3648	(59.4)	
65-80 years	298	(7.4)	159	(7.6)	457	(7.4)	
BMI, mean (SD)	49.3	(8.0)	49.2	(8.6)	49.3	(8.2)	0.01
BMI category, N (%)							
35-39 kg/m2	331	(8.2)	213	(10.1)	544	(8.9)	0.10
40-49 kg/m2	2129	(52.7)	1082	(51.5)	3211	(52.3)	
50-59 kg/m2	1176	(29.1)	560	(26.6)	1736	(28.3)	
60+ kg/m2	403	(10.0)	247	(11.8)	650	(10.6)	
Weight (kg), mean(SD)	126.1	(25.6)	125.6	(27.1)	125.9	(26.1)	0.02
Weight (kg), N (%)							
[45.4, 90)	150	(3.7)	97	(4.6)	247	(4.0)	0.07
[90, 135)	2584	(64.0)	1343	(64.0)	3927	(64.0)	
[135, 180)	1164	(28.9)	573	(27.3)	1737	(28.3)	
[180, 225)	125	(3.1)	83	(4.0)	208	(3.4)	
[225, 275]	12	(0.3)	4	(0.2)	16	(0.3)	
Missing	4	(0.1)	2	(0.1)	6	(0.1)	
Year of surgery, N (%)							
2005 -2009	694	(17.2)	39	(1.9)	733	(11.9)	0.76
2010	745	(18.5)	159	(7.6)	904	(14.7)	
2011	880	(21.8)	422	(20.1)	1302	(21.2)	
2012	719	(17.8)	459	(21.8)	1178	(19.2)	
2013	510	(12.6)	490	(23.3)	1000	(16.3)	
2014	402	(10.0)	426	(20.3)	828	(13.5)	
2015	89	(2.2)	107	(5.1)	196	(3.2)	
Hispanic Ethnicity, N (%)	983	(24.7)	614	(29.5)	1597	(26.3)	0.11
Missing Overall, N (%)	52	(1.3)	20	(1.0)	72	(1.2)	
Race, N (%)							
Asian	47	(1.4)	45	(2.5)	92	(1.8)	0.31
African-American	574	(16.4)	495	(27.9)	1069	(20.3)	

© 2020 McTigue KM et al. *JAMA Surgery.*

Multiple	1	(0.0)	3	(0.2)	4	(0.1)	
Caucasian	2661	(76.2)	1137	(64.2)	3798	(72.2)	
Pacific Islander	23	(0.7)	13	(0.7)	36	(0.7)	
Native American	38	(1.1)	13	(0.7)	51	(1.0)	
Other	147	(4.2)	66	(3.7)	213	(4.1)	
Missing Overall, N (%)	548	(13.6)	330	(15.7)	878	(14.3)	
HbA1c, mean (SD)	7.1	(1.1)	6.8	(0.9)	7.0	(1.1)	0.26
HbA1c category, N (%)							
<6.5	1174	(29.1)	691	(32.9)	1865	(30.4)	0.28
6.5-6.9	1087	(26.9)	690	(32.8)	1777	(28.9)	
7.0-7.9	1080	(26.7)	545	(25.9)	1625	(26.5)	
8.0-8.9	416	(10.3)	105	(5.0)	521	(8.5)	
9.0+	282	(7.0)	71	(3.4)	353	(5.8)	
Total Number of Diabetes Medications, mean (SD)	1.52	(1.1)	1.33	(1.1)	1.45	(1.1)	0.17
Total Number of Diabetes Medications, categorical							
0	866	(21.4)	612	(29.1)	1478	(24.1)	0.19
1	1078	(26.7)	545	(25.9)	1623	(26.4)	
2	1394	(34.5)	654	(31.1)	2048	(33.4)	
3	557	(13.8)	236	(11.2)	793	(12.9)	
4 +	144	(3.6)	55	(2.6)	199	(3.2)	
Diabetes Medications							
Biguanides	2534	(62.7)	1228	(58.4)	3762	(61.3)	0.09
GLP-1 receptor agonists	125	(3.1)	51	(2.4)	176	(2.9)	0.04
Insulins	1560	(38.6)	757	(36.0)	2317	(37.7)	0.05
Sulfonylureas	1283	(31.8)	532	(25.3)	1815	(29.6)	0.14
Thiazolidinediones	361	(8.9)	93	(4.4)	454	(7.4)	0.18
Other	265	(6.6)	127	(6.0)	392	(6.4)	0.02
Systolic BP, mean (SD)	129.5	(16.8)	130.6	(17.2)	129.9	(17.0)	0.06
Diastolic BP, mean (SD)	74.0	(10.8)	73.5	(11.6)	73.8	(11.1)	0.04
BP Category							
Norm	982	(24.6)	491	(23.5)	1473	(24.2)	0.05
Pre-hypertensive	1961	(49.1)	1002	(47.9)	2963	(48.7)	
Stage 1	829	(20.7)	470	(22.5)	1299	(21.3)	
Stage 2+	225	(5.6)	130	(6.2)	355	(5.8)	
Missing BP, N (%)	42	(1.0)	9	(0.4)	51	(0.8)	
Charlson-Elixhauser score category, mean (SD)	-0.1	(0.9)	-0.2	(1.0)	-0.1	(1.0)	0.05
Health Conditions, N (%)							
Anxiety	802	(19.9)	426	(20.3)	1228	(20.0)	0.01
Depression	1396	(34.6)	612	(29.1)	2008	(32.7)	0.12
Diabetes	3811	(94.4)	1879	(89.4)	5690	(92.7)	0.18
DVT	23	(0.6)	15	(0.7)	38	(0.6)	0.02

© 2020 McTigue KM et al. *JAMA Surgery.*

Dyslipidemia	2998	(74.2)	1543	(73.4)	4541	(74.0)	0.02
Eating Disorder	585	(14.5)	97	(4.6)	682	(11.1)	0.34
GERD	1711	(42.4)	753	(35.8)	2464	(40.1)	0.13
Hypertension	3258	(80.7)	1625	(77.3)	4883	(79.5)	0.08
Infertility	19	(0.5)	16	(0.8)	35	(0.6)	0.04
Kidney Disease	751	(18.6)	347	(16.5)	1098	(17.9)	0.06
NAFLD	1153	(28.6)	423	(20.1)	1576	(25.7)	0.20
Osteoarthritis	86	(2.1)	51	(2.4)	137	(2.2)	0.02
PCOS	160	(4.0)	95	(4.5)	255	(4.2)	0.03
PE	56	(1.4)	25	(1.2)	81	(1.3)	0.02
Psychotic Disorder	134	(3.3)	57	(2.7)	191	(3.1)	0.04
Sleep apnea	2370	(58.7)	1047	(49.8)	3417	(55.6)	0.18
Smoker	397	(9.8)	174	(8.3)	571	(9.3)	0.05
Substance Use Disorder	85	(2.1)	60	(2.9)	145	(2.4)	0.05
N of inpatient hospital days in year before surgery, mean (SD)	0.68	(9.4)	0.75	(8.7)	0.70	(9.2)	0.01
N of inpatient hospital days in categories							
zero	3749	(92.8)	1922	(91.4)	5671	(92.4)	0.06
1 to 7	232	(5.7)	149	(7.1)	381	(6.2)	
8 to 14	23	(0.6)	15	(0.7)	38	(0.6)	
15 or more	35	(0.9)	16	(0.8)	51	(0.8)	

© 2020 McTigue KM et al. *JAMA Surgery.*

eTable 5: Relapse findings using different approaches to censoring at the occurrence of inpatient hospital stays

	Adjusted HR (95% CI)		P-Value	Adjusted proportion relapsed at 1, 3, and 5 years (95% CI)									
					1 year			3 years			5 years		
Censoring of all patients at the first inpatient stays	0.74	(0.65, 0.84)	<0.001	RYGB	8.0	(7.0,	9.0)	20.1	(17.8,	22.3)	33.6	(29.2,	37.8)
				SG	10.6	(9.1,	12.1)	26.1	(22.8,	29.2)	42.5	(36.6,	47.8)
No censoring of patients at inpatient stays	0.78	(0.70, 0.87)	<0.0001	RYGB	9.7	(8.7,	10.7)	23.5	(21.4,	25.6)	36.7	(33.1,	40.2)
				SG	12.3	(10.8,	13.7)	29.1	(26.0,	32.1)	44.4	(39.6,	48.9)

© 2020 McTigue KM et al. *JAMA Surgery.*

eTable 6: Relapse findings restricting the sample to integrated health systems[a]

Adjusted HR (95% CI)	P-Value	Adjusted proportion relapsed at 1, 3, and 5 years (95% CI)			
			1 year	3 years	5 years
0.68 (0.60, 0.76)	<0.0001	RYGB	8.8 (7.7, 9.8)	22.2 (20.0, 24.4)	34.8 (31.0, 38.3)
		SG	12.7 (11.0, 14.4)	31.1 (27.6, 34.4)	46.9 (41.6, 51.7)

[a]Integrated health systems include those systems that integrate health care delivery with health insurance coverage: Kaiser Permanente (Southern California, Northwest, Colorado, Mid-Atlantic, and Washington), Health Partners, Geisinger Health System, and Marshfield Clinic

© 2020 McTigue KM et al. *JAMA Surgery.*

eFigure 2. Unadjusted proportion of patients with well-controlled (<6.5%; left panel) or poorly controlled (≥8%) HbA1c at the different follow-up periods for each of the surgical groups. Error bars indicate 95% confidence intervals.

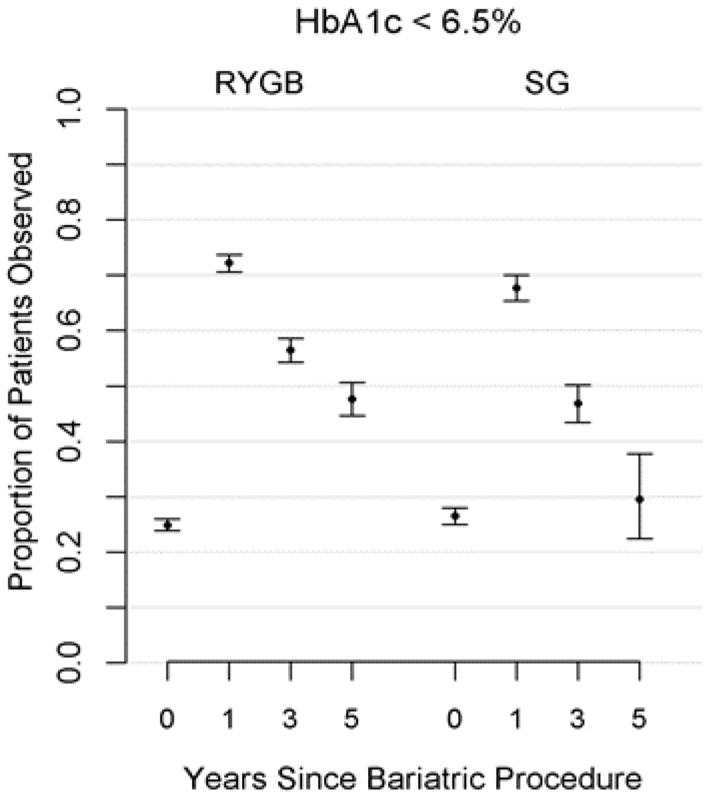

© 2020 McTigue KM et al. *JAMA Surgery.*

eTable 7: Effect of subgroup factors on T2DM remission rates by procedure. Hazard ratios compare RYGB versus SG effectiveness for T2DM remission, by category of DiaRem Score.*

DiaRem Score	HR	95% Confidence Interval	P Value	Interaction P-Value
0 - 2	1.009	(0.880, 1.158)	0.8943	0.0460
3 - 7	1.048	(0.955, 1.151)	0.3229	
8 - 12	1.021	(0.859, 1.215)	0.8100	
13 - 17	1.237	(1.102, 1.389)	0.0003	
18 - 22	1.500	(0.989, 2.275)	0.0561	

*Score indicates pre-operative prediction of T2DM remission following RYGB Surgery, where a higher score indicates lower probability of T2DM remission: 0–2 (88%–99%), 3–7 (64%–88%), 8–12 (23%–49%), 13–17 (11%–33%), 18–22 (2%–16%).

© 2020 McTigue KM et al. *JAMA Surgery.*

eTable 8. Estimated percentage of T2DM remission by type of procedure and DiaRem Score

Procedure	DiaRem	Time Point	N	Remission %	95% CI	
RYGB	0-2	1 year	693	58.4	50.7	64.9
		3 years		83.7	76.8	88.5
		5 years		85.5	78.9	90.0
	3-7	1 year	1909	65.8	60.2	70.5
		3 years		89.1	85.1	92.0
		5 years		90.5	86.8	93.2
	8-12	1 year	686	51.4	46.6	55.8
		3 years		77.4	72.6	81.4
		5 years		79.6	74.8	83.4
	13-17	1 year	1847	55.8	49.7	61.2
		3 years		81.5	75.8	85.9
		5 years		83.4	77.9	87.6
	18-22	1 year	293	61.8	49.7	71.0
		3 years		86.3	75.8	92.2
		5 years		88.0	77.9	93.4
SG	0-2	1 year	420	58.1	49.9	64.9
		3 years		83.4	76.0	88.5
		5 years		85.2	78.1	90.0
	3-7	1 year	1084	64.0	58.0	69.2
		3 years		87.9	83.3	91.2
		5 years		89.5	85.1	92.5
	8-12	1 year	327	50.6	44.4	56.2
		3 years		76.7	70.2	81.8
		5 years		78.9	72.5	83.8
	13-17	1 year	1006	48.3	42.1	53.9
		3 years		74.4	67.7	79.8
		5 years		76.6	70.0	81.8
	18-22	1 year	98	47.3	30.7	60.0
		3 years		73.4	53.1	84.9
		5 years		75.6	55.4	86.7

**Score indicates pre-operative prediction of T2DM remission following RYGB Surgery, where a higher score indicates lower probability of T2DM remission: 0–2 (88%–99%), 3–7 (64%–88%), 8–12 (23%–49%), 13–17 (11%–33%), 18–22 (2%–16%).*

Supplemental Appendix 2 – Comparing Adjustable Gastric Banding to Roux-en-Y Gastric Bypass and Sleeve Gastrectomy

AGB eText	Description of results comparing AGB vs. RYGB and AGB vs. SG
AGB eTable 1	Description of sample including AGB, RYGB, and SG at baseline [frequency (%) or mean (SD)]
AGB eTable 2	Adjusted hazard ratios comparing time to remission since surgery and time to relapse since remission for comparisons of AGB vs RYGB and AGB vs SG
AGB eFigure 1	Cumulative incidence rates of T2DM remission among all AGB patients in the PBS T2DM cohort compared with RYGB and SG (left panel); and cumulative incidence rates of relapse among all AGB patients who experienced an initial remission (right panel) across 5 years in the PBS cohort compared with RGYB and SG.
AGB eFigure 2	Adjusted Absolute difference in HbA1c for AGB, RYGB, and SG procedures over 5 years of follow-up.
AGB eTable 3	Comparative Effectiveness of AGB vs RYGB and AGB vs SG for Absolute Differences in HbA1c among Adults at 1, 3, and 5 Years Follow-up
AGB eTable 4	Adjusted hazard ratios comparing time to remission for AGB vs RYGB and AGB vs SG, using a 9-month (top panel) or 12-month (bottom panel) cut point in censoring*
AGB eTable 5	Adjusted hazard ratios comparing time to remission for comparisons of AGB vs RYGB and AGB vs. SG, with sample restricted to integrated health system
AGB eTable 6	Relapse findings comparing AGB vs RYGB and AGB vs SG using different approaches to censoring at the occurrence of inpatient hospital stays
AGB eTable 7	Relapse findings comparing AGB vs RYGB and AGB vs SG restricting the sample to integrated health systems
AGB eFigure 3	Unadjusted proportion of patients with well-controlled (<6.5%; left panel) or poorly controlled (≥8%) HbA1c at the different follow-up periods for AGB, RYGB and SG.

AGB eText. Description of results comparing AGB vs. RYGB and AGB vs. SG

Our original protocol called for three pairwise comparisons: RYGB vs SG; AGB vs RYGB; and AGB vs. SG. The RYGB and SG results have been reported in the main manuscript because they are the most relevant procedures performed worldwide today. At the time of the funding of our project, the adjustable gastric banding (AGB) procedure was still widely performed, although it was already waning in its use. Nevertheless, our study stakeholders encouraged us to compare RYGB and SG outcomes to AGB to help inform patients and health care professionals of the 5-year comparative safety of these three procedures. This AGB Appendix includes our original analyses that have AGB comparisons. Where data are presented for RYGB and SG, they are *only in comparison to AGB.* That is, the estimates for RYGB and SG that are reported are those generated in analyses of data from only those data contributing sites in the PBS study that performed AGB procedures as well as RYGB or SG procedures (the comparator). Thus, sample sizes for RYGB and SG in these tables may differ from the main manuscript.

Overall, the PBS cohort included many fewer AGB procedures among patients with type 2 diabetes (n=308; see AGB eTable 1). As with prior studies, patients who had AGB were generally older, with lower baseline BMI, and fewer AGB patients were on insulin at the time of surgery, compared with RYGB and SG patients. Otherwise baseline characteristics were similar.

The AGB eTable 2 shows the main study result, which is that patients undergoing RYGB had 2.19 times the rate of remission as those undergoing AGB [1.89, 2.53]. The SG versus AGB comparison showed similar results [HR 1.85 (1.53, 2.25)]. Among AGB patients, less than 40% experienced T2DM remission by one year of follow-up and cumulative remission rates were 65% or less over five years of follow-up (see also AGB eFigure 1). RYGB and SG each showed nearly 70% lower relapse rates than AGB (HRs of 0.32-0.33). Cumulative rates of T2DM relapse were highest for AGB patients; relapse rates were also higher among SG patients than RYGB patients.

Consistent with our main analyses reported in the manuscript, sensitivity analyses (AGB eTables 4, 5, 6, and 7) did not substantially change our findings. Sensitivity analyses requiring 9-month and 12-month timeframes without a diabetes medication prescription to define T2DM remission produced similar results to the 6-month timeframe used in the primary analysis, although the differences between SG and RGB were not always statistically significant (AGB eTable 4). Additional sensitivity analyses restricted to integrated health plans yielded qualitatively similar results to the primary comparative analyses, despite slightly higher cumulative remission rates for SG and RYGB, and slightly lower rates for AGB (eTable 5). Different approaches to censoring had a minimal impact (eTable 6), while among integrated health systems, AGB showed somewhat higher relapse rates across all participating sites (eTable 7).

The three bariatric procedures had differential effects on T2DM control, with patients who underwent RYGB experiencing the largest and most-sustained HbA1c reductions, followed by SG and then AGB (AGB eFigure 2; AGB eTable 3). RYGB and SG consistently out-performed banding in terms of HbA1c change.

The conclusion is that AGB patients showed significantly lower rates of T2DM remission, higher rates of relapse, and worse glycemic control than either RYGB or SG procedures over the 5 year study time frame. These results further support the decline in use of the AGB procedure.

AGB eTable 1. Description of sample including AGB, RYGB, and SG at baseline [frequency (%) or mean (SD)]

	AGB		RYGB		SG		Overall	
N (row %)	308	(3.1)	6233	(62.2)	3477	(34.7)	10018	(100.0)
Female, N (%)	212	(68.8)	4576	(73.4)	2475	(71.2)	7263	(72.5)
Age, mean (SD)	52.6	(10.8)	49.9	(10.4)	49.7	(10.8)	49.9	(10.6)
Age category, N (%)								
20-44 years	72	(23.4)	1929	(31.0)	1117	(32.1)	3118	(31.1)
45-64 years	192	(62.3)	3819	(61.3)	2065	(59.4)	6076	(60.7)
65-79 years	44	(14.3)	485	(7.8)	295	(8.5)	824	(8.2)
BMI, mean (SD)	45.9	(7.0)	49.0	(8.2)	49.0	(8.6)	48.9	(8.3)
BMI category, N (%)								
35-39 kg/m^2	61	(19.8)	638	(10.2)	386	(11.1)	1085	(10.8)
40-49 kg/m^2	180	(58.4)	3250	(52.1)	1781	(51.2)	5211	(52.0)
50-59 kg/m^2	55	(17.9)	1739	(27.9)	917	(26.4)	2711	(27.1)
60+ kg/m^2	12	(3.9)	606	(9.7)	393	(11.3)	1011	(10.1)
Weight (kg), mean (SD)	120.9	(24.1)	125.7	(25.6)	125.6	(27.1)	125.5	(26.1)
Year of surgery, N (%)								
2005 -2009	74	(24.0)	969	(15.6)	53	(1.5)	1096	(10.9)
2010	76	(24.7)	1049	(16.8)	216	(6.2)	1341	(13.4)
2011	76	(24.7)	1250	(20.1)	570	(16.4)	1896	(18.9)
2012	52	(16.9)	1037	(16.6)	657	(18.9)	1746	(17.4)
2013	19	(6.2)	798	(12.8)	743	(21.4)	1560	(15.6)
2014	10	(3.3)	744	(11.9)	840	(24.2)	1594	(15.9)
2015	1	(0.3)	386	(6.2)	398	(11.5)	785	(7.8)
Hispanic Ethnicity, N (%)	32	(10.7)	1407	(22.9)	971	(28.3)	2410	(24.4)
Missing Overall, N (%)	10	(3.3)	91	(1.5)	42	(1.2)	143	(1.4)
Race, N (%)								
Asian	2	(0.7)	86	(1.6)	69	(2.4)	157	(1.8)
African-American	90	(32.1)	900	(16.6)	800	(27.3)	1790	(20.7)
Multiple	0	(0.0)	3	(0.1)	5	(0.2)	8	(0.1)
Caucasian	175	(62.5)	4136	(76.2)	1904	(64.9)	6215	(71.9)
Pacific Islander	0	(0.0)	32	(0.6)	19	(0.7)	51	(0.6)
Native American	1	(0.4)	49	(0.9)	21	(0.7)	71	(0.8)
Other	12	(4.3)	225	(4.1)	117	(4.0)	354	(4.1)
Missing Overall, N (%)	28	(9.1)	802	(12.9)	542	(15.6)	1372	(13.7)
HbA1c, mean (SD)	7.3	(1.3)	7.3	(1.3)	7.1	(1.2)	7.2	(1.3)
HbA1c category, N (%)								
<6.5%	77	(25.0)	1554	(24.9)	922	(26.5)	2553	(25.5)
6.5-6.9%	71	(23.1)	1408	(22.6)	951	(27.4)	2430	(24.3)
7.0-7.9%	85	(27.6)	1738	(27.9)	995	(28.6)	2818	(28.1)
8.0-8.9%	39	(12.7)	834	(13.4)	354	(10.2)	1227	(12.3)
9.0%+	36	(11.7)	699	(11.2)	255	(7.3)	990	(9.9)

AGB eTable 1. Description of sample including AGB, RYGB, and SG at baseline [frequency (%) or mean (SD)]

	AGB		RYGB		SG		Overall	
Total Number of Diabetes Medications, mean (SD)	1.53	(1.1)	1.70	(1.1)	1.60	(1.1)	1.66	(1.1)
Total Number of Diabetes Medications, categorical								
0	60	(19.5)	1096	(17.6)	747	(21.5)	1903	(19.0)
1	90	(29.2)	1354	(21.7)	772	(22.2)	2216	(22.1)
2	108	(35.1)	2447	(39.3)	1266	(36.4)	3821	(38.1)
3	38	(12.3)	1048	(16.8)	546	(15.7)	1632	(16.3)
4 to 7	12	(3.9)	288	(4.6)	146	(4.2)	446	(4.5)
Diabetes Medications, N (%)								
Biguanides	193	(62.7)	4109	(65.9)	2237	(64.3)	6539	(65.3)
GLP-1 receptor agonists	22	(7.1)	278	(4.5)	148	(4.3)	448	(4.5)
Insulins	110	(35.7)	3047	(48.9)	1645	(47.3)	4802	(47.9)
Sulfonylureas	90	(29.2)	2054	(33.0)	1058	(30.4)	3202	(32.0)
Thiazolidinediones	33	(10.7)	609	(9.8)	198	(5.7)	840	(8.4)
Other	22	(7.1)	477	(7.7)	260	(7.5)	759	(7.6)
Systolic BP, mean (SD)	129.5	(17.7)	130.1	(17.0)	131.3	(17.5)	130.5	(17.2)
Diastolic BP, mean (SD)	76.2	(12.7)	73.8	(10.9)	73.5	(11.6)	73.7	(11.2)
Blood Pressure Category[a]								
Normotensive	71	(23.5)	1473	(23.9)	779	(22.6)	2323	(23.4)
Pre-hypertensive	145	(48.0)	2991	(48.5)	1626	(47.1)	4762	(48.0)
Stage 1 Hypertension	64	(21.2)	1320	(21.4)	812	(23.5)	2196	(22.1)
Stage 2+ Hypertension	22	(7.3)	379	(6.2)	236	(6.8)	637	(6.4)
Missing BP, N (%)	6	(2.0)	70	(1.1)	24	(0.7)	100	(1.0)
Charlson/Elixhauser score, N (%)								
≤-1	112	(36.4)	2454	(39.4)	1465	(42.1)	4031	(40.2)
0	124	(40.3)	2384	(38.3)	1271	(36.6)	3779	(37.7)
≥1	72	(23.4)	1395	(22.4)	741	(21.3)	2208	(22.0)
Health Conditions, N (%)								
Hypertension	257	(83.4)	5113	(82.0)	2729	(78.5)	8099	(80.8)
Dyslipidemia	244	(79.2)	4775	(76.6)	2659	(76.5)	7678	(76.6)
Sleep apnea	169	(54.9)	3607	(57.9)	1740	(50.0)	5516	(55.1)
GERD	129	(41.9)	2609	(41.9)	1264	(36.4)	4002	(40.0)
Depression	90	(29.2)	2157	(34.6)	1053	(30.3)	3300	(32.9)
NAFLD	66	(21.4)	1914	(30.7)	730	(21.0)	2710	(27.1)
Anxiety	50	(16.2)	1274	(20.4)	734	(21.1)	2058	(20.5)
Kidney Disease	57	(18.5)	1268	(20.3)	670	(19.3)	1995	(19.9)
Eating Disorder	19	(6.2)	969	(15.6)	231	(6.6)	1219	(12.2)
Smoker	20	(6.5)	582	(9.3)	276	(7.9)	878	(8.8)
Pulmonary Embolism	10	(3.3)	257	(4.1)	147	(4.2)	414	(4.1)

AGB eTable 1. Description of sample including AGB, RYGB, and SG at baseline [frequency (%) or mean (SD)]

	AGB		RYGB		SG		Overall	
Psychotic Disorder	8	(2.6)	197	(3.2)	96	(2.8)	301	(3.0)
Substance Use Disorder	9	(2.9)	143	(2.3)	102	(2.9)	254	(2.5)
	AGB		RYGB		SG		Overall	
Osteoarthritis, lower limb	5	(1.6)	148	(2.4)	93	(2.7)	246	(2.5)
PCOS	2	(0.7)	87	(1.4)	39	(1.1)	128	(1.3)
DVT	2	(0.7)	38	(0.6)	28	(0.8)	68	(0.7)
Infertility	1	(0.3)	29	(0.5)	29	(0.8)	59	(0.6)
N of inpatient hospital days in year before surgery, mean (SD)	0.69	(4.5)	0.67	(8.0)	0.83	(8.0)	0.73	(7.9)
N of inpatient hospital days in year before surgery, Categories								
Zero	284	(92.2)	5758	(92.4)	3156	(90.8)	9198	(91.8)
1 to 7	17	(5.5)	373	(6.0)	253	(7.3)	643	(6.4)
8 to 14	3	(1.0)	45	(0.7)	36	(1.0)	84	(0.8)
15 or more	4	(1.3)	57	(0.9)	32	(0.9)	93	(0.9)

Baseline = measured in the year prior to surgery; AGB = adjustable gastric banding; RYGB = Roux-en-Y gastric bypass; SG = sleeve gastrectomy; BMI = body mass index (kg/m^2); SD = standard deviations; BP = blood pressure in year prior to surgery; Health Conditions were identified by 1+ ICD-9 or SNOMED diagnosis code in the year prior to surgery; NAFLD = non-alcoholic fatty liver disease; GERD = gastroesophageal reflux disease; PCOS = polycystic ovarian syndrome; DVT = deep vein thrombosis; [a]Normotensive: SBP<120 and DBP<80; Pre-hypertensive : SBP 120-129 and DBP<80; Stage 1: SBP 130-139 and DBP 80-89; Stage 1 Hypertension: SBP ≧ 140 and DBP ≥ 90; Stage 2+ Hypertension: SBP ≥ 180 and DBP ≥ 120).

AGB eTable 2. Adjusted hazard ratios comparing time to remission since surgery and time to relapse since remission for comparisons of AGB vs RYGB and AGB vs SG.

T2DM Remission	Adjusted HR[a] (95% CI)	P-Value	Estimated cumulative percentage of patients with T2DM who had experienced an initial remission (95% CI) at different times of follow-up			
				1 year	3 years	5 years
RYGB vs AGB	2.19 (1.89, 2.53)	<0.0001	RYGB	57.8 (56.2, 59.3)	83.5 (82.1, 84.7)	85.4 (83.9, 86.7)
			AGB	32.6 (27.8, 37.1)	56.1 (49.4, 61.9)	58.5 (51.6, 64.3)
SG vs AGB	1.85 (1.53, 2.25)	<0.0001	SG	56.0 (53.7, 58.3)	83.3 (81.1, 85.3)	86.0 (83.1, 88.3)
			AGB	35.8 (29.2, 41.7)	61.9 (53.0, 69.1)	65.3 (55.9, 72.7
**T2DM Relapse**	**Adjusted HR[b] (95% CI)**	**P-Value**	**Estimated cumulative percentage of patients with initial T2DM remission who had experienced relapse (95% CI) at different times of follow-up**			
				1 year	3 years	5 years
RYGB vs AGB	0.32 (0.26, 0.41)	<0.0001	RYGB	9.8 (8.7, 11.0)	22.7 (20.6, 24.8)	34.3 (30.8, 37.5)
			AGB	27.3 (20.6, 33.4)	54.8 (43.8, 63.7)	72.5 (60.3, 81.0)
SG vs AGB	0.33 (0.24, 0.44)	<0.0001	SG	9.8 (8.1, 11.4)	27.2 (23.2, 31.1)	43.3 (34.6, 50.8)
			AGB	26.9 (18.4, 34.6)	62.1 (46.5, 73.2)	82.3 (64.3, 91.2)

[a]Remission of diabetes defined as HbA1c <6.5% after 6 months without any prescription order for a diabetes medication; Covariates included: age, sex, race, Hispanic ethnicity, BMI, HbA1c, blood pressure, days from BMI measurement to baseline, number of inpatient hospital days in the year prior to surgery, number of diabetes medications excluding insulin, insulin use, Charlson/Elixhauser comorbidity score, year of procedure, having a code for diabetes, smoking, having a code for other comorbidities (hypertension, dyslipidemia, sleep apnea, osteoarthritis, NAFLD, GERD, depression, anxiety, eating disorder, substance use, psychosis, kidney disease, infertility, polycystic ovaries, deep vein thrombosis, pulmonary embolism), having codes for specific diabetes medications (biguanides, GLP-1 agonists, sulfonylureas, thiazolidinediones, and other), site and propensity score deciles.

[b] Relapse of diabetes defined as occurrence of any HbA1c ≥6.5% and/or prescription order for a diabetes medication Covariates included: age, sex, race, Hispanic ethnicity, BMI, HbA1c, blood pressure, days from BMI measurement to baseline, number of inpatient hospital days in the year prior to surgery, number of diabetes medications excluding insulin, insulin use, Charlson/Elixhauser comorbidity score, year of procedure, having a code for diabetes, smoking, having a code for other comorbidities (hypertension, dyslipidemia, sleep apnea, osteoarthritis, NAFLD, GERD, depression, anxiety, eating disorder, substance use, psychosis, kidney disease, infertility, polycystic ovaries, deep vein thrombosis,

pulmonary embolism), having codes for specific diabetes medications (biguanides, GLP-1 agonists, sulfonylureas, thiazolidinediones, and other), site and propensity score deciles

AGB eFigure 1. Cumulative incidence rates of T2DM remission among all AGB patients in the PBS T2DM cohort compared with RYGB and SG (left panel); and cumulative incidence rates of relapse among all AGB patients who experienced an initial remission (right panel) across 5 years in the PBS cohort compared with RGYB and SG.

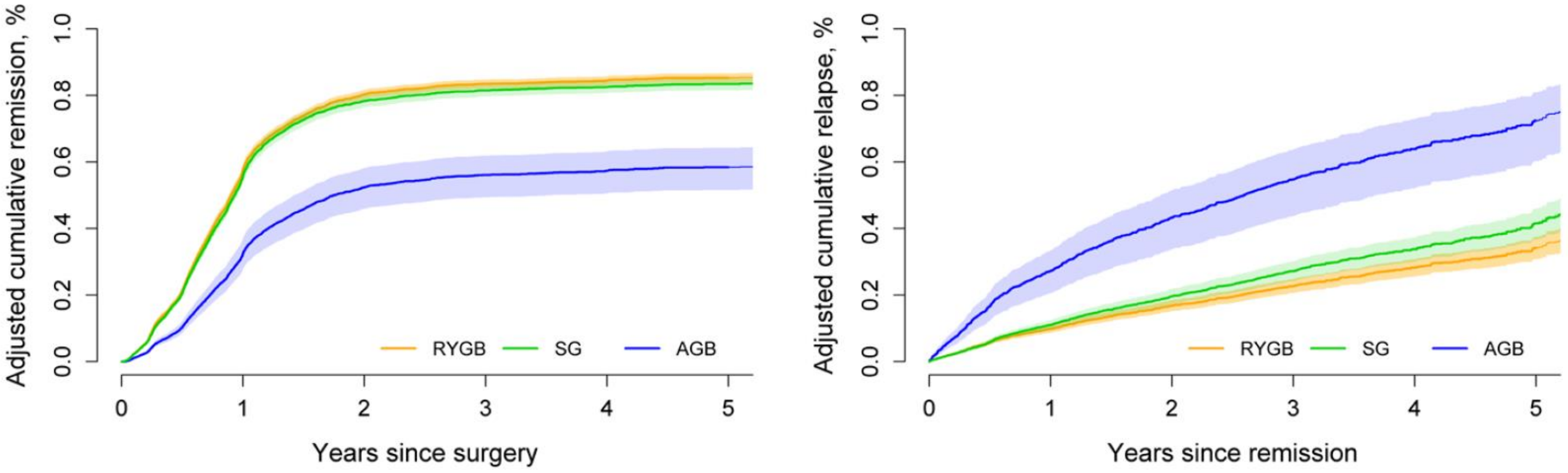

AGB eTable 3. Comparative Effectiveness of AGB vs RYGB and AGB vs SG for Absolute Difference[a] in HbA1c among Adults at 1, 3, and 5 Years Follow-up

Groups	N	HbA1c Mean Difference* (95% CI)	Groups	N	HbA1c Mean Difference[a] (95% CI)
**SGB**	280	-0.55 (-0.65, -0.45)	**AGB**	277	-0.40 (-0.51, -0.29)
**RYGB**	5,367	-1.20 (-1.23, -1.18)	**SG**	2,547	-0.84 (-0.88, -0.81)
		-0.66 (-0.76, -0.56)			-0.44 (-0.56, -0.32)
		0.000			0.000
**AGB**	280	-0.20 (-0.38, -0.03)	**AGB**	277	-0.16 (-0.36, 0.03)
**RYGB**	5,367	-0.99 (-1.04, -0.95)	**SG**	2,547	-0.52 (-0.60, -0.45)
		-0.79 (-0.97, -0.61)			-0.36 (-0.57, -0.15)
		0.000			0.001
**AGB**	280	0.00 (-0.30, 0.29)	**AGB**	277	0.09 (-0.23, 0.41)
**RYGB**	5,367	-0.85 (-0.92, -0.77)	**SG**	2,547	-0.33 (-0.49, -0.16)
		-0.84 (-1.15, -0.54)			-0.42 (-0.78, -0.05)
		0.000			0.024

[a]Difference = baseline value – longitudinal value; model adjusted for age, sex, race, Hispanic ethnicity, BMI, HbA1c, blood pressure, number of inpatient hospital days in the year prior to surgery, number of diabetes medications excluding insulin, insulin use, Charlson/Elixhauser comorbidity score, year of procedure, days from HbA1c measurement to baseline, having a code for diabetes, smoking, having a code for other comorbidities (hypertension, dyslipidemia, sleep apnea, osteoarthritis, NAFLD, GERD, depression, anxiety, eating disorder, substance use, psychosis, kidney disease, infertility, polycystic ovaries, deep vein thrombosis, pulmonary embolism), having codes for specific diabetes medications (biguanides, GLP-1 agonists, sulfonylureas, thiazolidinediones, and other), site and propensity score deciles

AGB eFigure 2. Adjusted Absolute difference in HbA1c for AGB, RYGB, and SG procedures over 5 years of follow-up.

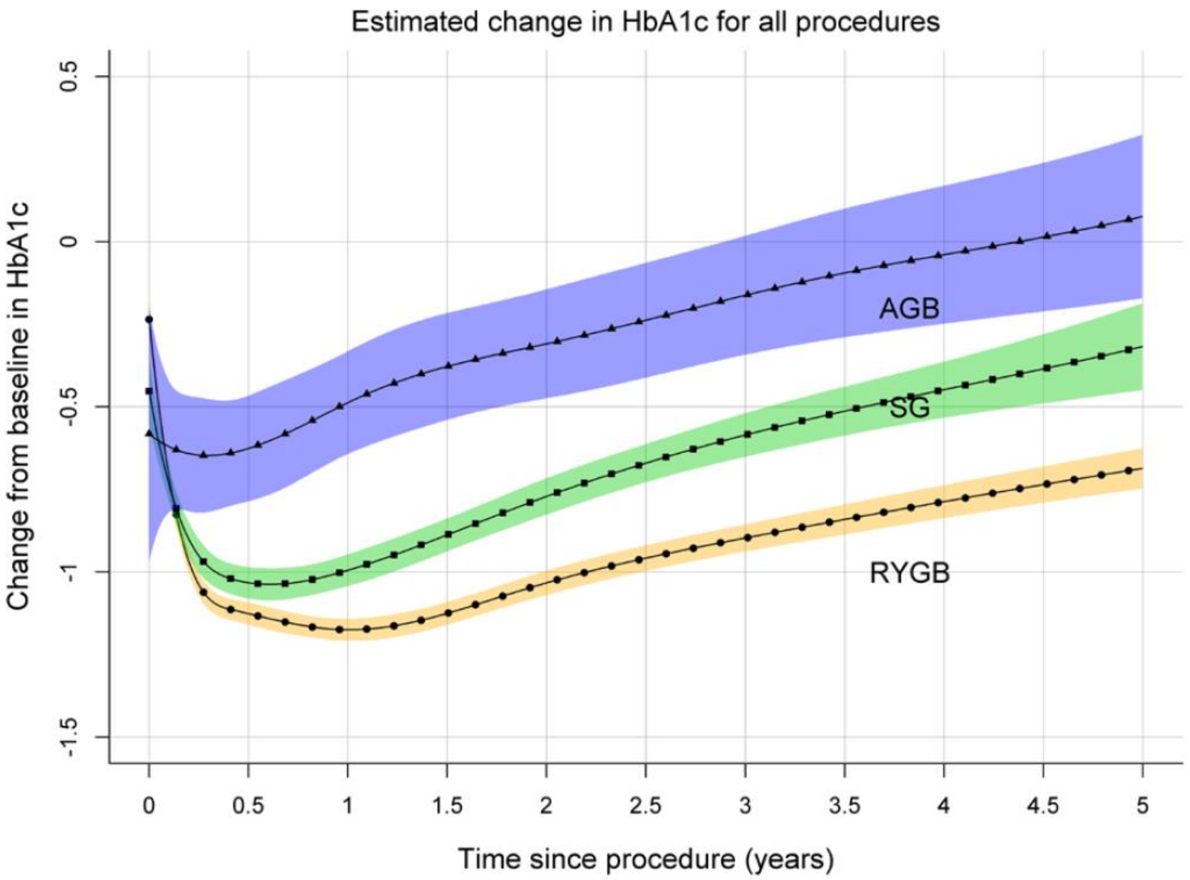

AGB eTable 4: Adjusted hazard ratios comparing time to remission for AGB vs RYGB and AGB vs SG, using a 9-month (top panel) or 12-month (bottom panel) cut point In censoring*

**9-Months**	**Adjusted HR* (95% CI)**	**P-Value**	**Estimated cumulative percentage of patients with T2DM who had experienced remission (95% CI) at different times of follow-up**			
				**1 year**	**3 years**	**5 years**
RYGB vs SG	1.00 (0.94, 1.06)	0.989	RYGB	40.2 (38.7, 41.6)	76.9 (75.3, 78.4)	79.3 (77.7, 80.9)
			SG	40.2 (38.3, 42.0)	76.9 (74.8, 78.9)	79.4 (77.2, 81.3)
RYGB vs AGB	2.13 (1.82, 2.49)	<0.0001	RYGB	39.7 (38.2, 41.2)	77 (75.3, 78.5)	79.2 (77.5, 80.8)
			AGB	21.2 (17.5, 24.7)	49.9 (43, 55.9)	52.2 (45.2, 58.4)
SG vs AGB	2.00 (1.62, 2.48)	<0.0001	SG	39.3 (37.1, 41.5)	77.3 (74.7, 79.6)	81.0 (78.0, 83.5)
			AGB	22.1 (17.2, 26.7)	52.3 (43.1, 60)	56.3 (46.6, 64.2)
**12-Months**	**Adjusted HR† (95% CI)**	**P-Value**	**Estimated cumulative percentage of patients with T2DM who had experienced remission (95% CI) at different times of follow-up**			
				**1 year**	**3 years**	**5 years**
RYGB vs AGB	2.4 (2, 2.9)	<0.0001	RYGB	17.8 (16.7, 19)	67.2 (65.2, 69.1)	70.7 (68.6, 72.7)
			AGB	7.7 (6.1, 9.3)	36.6 (30.3, 42.4)	39.5 (32.8, 45.6)
SG vs AGB	2.5 (2.0, 3.2)	<0.0001	SG	17.3 (15.7, 18.8)	65.9 (62.9, 68.6)	70.8 (67.3, 74)
			AGB	7.2 (5.2, 9.2)	34.7 (26.8, 41.7)	38.6 (29.9, 46.2)

**AGB eTable 5: Adjusted hazard ratios comparing time to remission for comparisons of AGB vs RYGB and AGB vs. SG, with sample restricted to integrated health system[a]**						
**Comparison**	**Adjusted HR* (95% CI)**	**P-Value**	**Estimated cumulative percentage of patients with T2DM who had experienced remission (95% CI) at different times of follow-up**			
				**1 year**	**3 years**	**5 years**
RYGB vs AGB	2.46 (2.02, 2.99)	<0.0001	RYGB	61.0 (59.3, 62.7)	84.3 (82.8, 85.7)	86.1 (84.5, 87.5)
			AGB	31.8 (25.5, 37.6)	52.9 (43.9, 60.4)	55.1 (46.0, 62.7)
SG vs AGB	2.12 (1.65, 2.73)	<0.0001	SG	61.6 (58.9, 64.1)	85.9 (83.7, 87.9)	88.5 (83.5, 91.3)
			AGB	36.2 (27.3, 44.1)	60.3 (48.2, 69.6)	64.0 (49.2, 74.2)

[a]Integrated health systems include those systems that integrate health care delivery with health insurance coverage: Kaiser Permanente (Southern California, Northwest, Colorado, Mid-Atlantic States, and Washington), Health Partners, Geisinger Health System, and Marshfield Clinic

AGB eTable 6: Relapse findings comparing AGB vs RYGB and AGB vs SG using different approaches to censoring at the occurrence of inpatient hospital stays

**Censoring at all patients at the first inpatient stays**													
	**Adjusted HR (95% CI)**		**P-Value**	**Adjusted proportion relapsed at 1, 3, and 5 years (95% CI)**									
					**1 year**			**3 years**			**5 years**		
RYGB vs AGB	0.28	(0.22, 0.36)	<0.0001	RYGB	9.4	(8.2,	10.5)	21.2	(18.9,	23.4)	33.5	(29.3,	37.4)
				AGB	29.5	(22.0,	36.3)	57.2	(45.1,	66.5)	76.5	(63.2,	85.0)
SG vs AGB	0.34	(0.24, 0.46)	<0.0001	SG	9.7	(8.0,	11.5)	26.9	(22.5,	31.0)	43.6	(33.1,	52.3)
				AGB	26.3	(17.3,	34.3)	60.7	(43.7,	72.5)	81.8	(60.8,	91.5)
**No Censoring of patients at inpatient stays**													
	**Adjusted HR (95% CI)**		**P-Value**	**Adjusted proportion relapsed at 1, 3, and 5 years (95% CI)**									
					**1 year**			**3 years**			**5 years**		
RYGB vs AGB	0.33	(0.26, 0.41)	<0.0001	RYGB	11.2	(10.0,	12.4)	25.1	(22.9,	27.3)	37.5	(34.1,	40.8)
				AGB	30.3	(23.2,	36.7)	58.5	(47.5,	67.2)	76.1	(64.4,	83.9)
SG vs AGB	0.34	(0.26, 0.46)	<0.0001	SG	11.0	(9.2,	12.8)	29.4	(25.2,	33.3)	47.8	(38.6,	55.6)
				AGB	28.8	(20.0,	36.6)	63.7	(48.4,	74.4)	85.0	(68.2,	92.9)

AGB eTable 7: Relapse findings comparing AGB vs RYGB and AGB vs SG restricting the sample to integrated health systems[a]						
	Adjusted HR (95% CI)	P-Value	Adjusted proportion relapsed at 1, 3, and 5 years (95% CI)			
				1 year	3 years	5 years
RYGB vs SG	0.68 (0.60, 0.76)	<0.0001	RYGB	8.8 (7.7, 9.8)	22.2 (20.0, 24.4)	34.8 (31.0, 38.3)
			SG	12.7 (11.0, 14.4)	31.1 (27.6, 34.4)	46.9 (41.6, 51.7)
RYGB vs AGB	0.26 (0.20, 0.34)	<0.0001	RYGB	10.3 (9.0, 11.5)	23.9 (21.5, 26.2)	36.2 (32.5, 39.7)
			AGB	34.2 (24.4, 42.7)	65.1 (50.6, 75.4)	82.3 (68.1, 90.2)
SG vs AGB	0.31 (0.22, 0.44)	<0.0001	SG	11.2 (9.3, 13.1)	31.3 (26.8, 35.5)	49.7 (39.0, 58.5)
			AGB	31.9 (20.1, 42.0)	70.2 (50.4, 82.1)	89.1 (68.0, 96.3)

[a]Integrated health systems include those systems that integrate health care delivery with health insurance coverage: Kaiser Permanente (Southern California, Northwest, Colorado, Mid-Atlantic, and Washington), Health Partners, Geisinger Health System, and Marshfield Clinic

AGB eFigure 3. Unadjusted proportion of patients with well-controlled (<6.5%; left panel) or poorly controlled (≥8%) HbA1c at the different follow-up periods for AGB, RYGB and SG. Error bars indicate 95% confidence intervals.

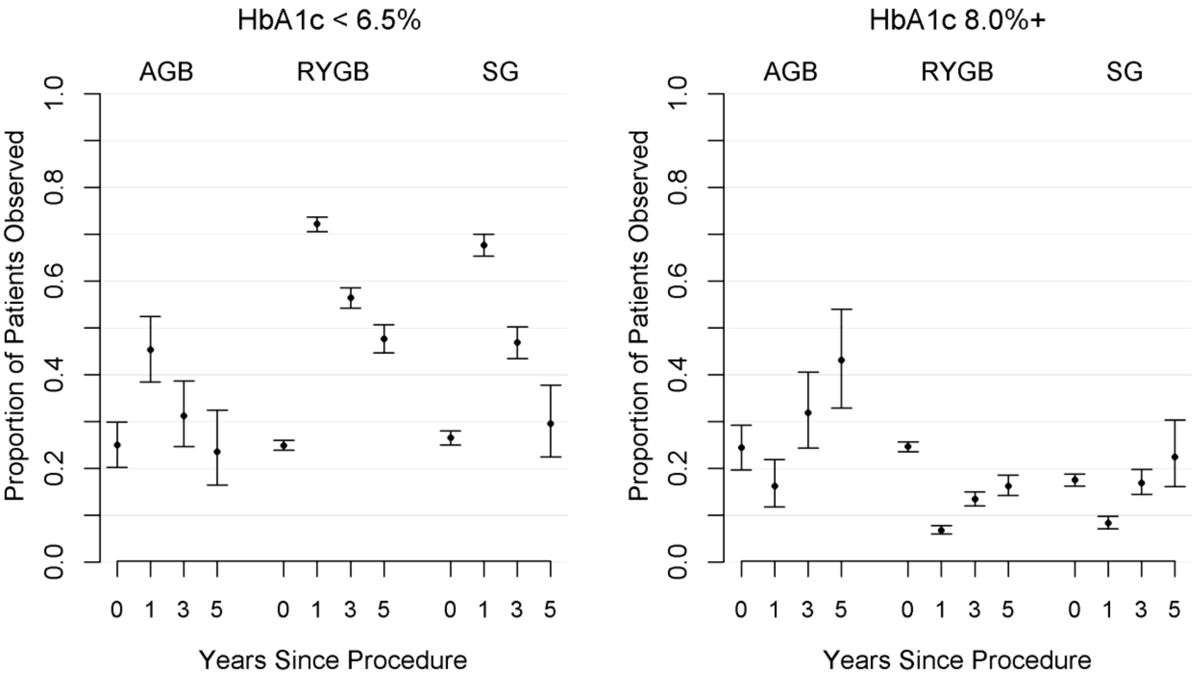

Supplement: Supplement. — eTable 1. List of Clinical Data Research Networks and Sites participating in the PCORnet Bariatric Study eFigure 1. Flow diagram for cohort selection eAppendix. Eligibility criteria for PBS Diabetes Analyses eTable 2. Remission findings using 9- and 12-month censoring options eTable 3. Remission findings with sample restricted to IHPs eTable 4. Descriptive features of patients examined in the relapse analysis eTable 5. Relapse findings using different approaches to censoring eTable 6. Relapse findings restricted to Integrated Health Plans eFigure 2. Unadjusted proportion of patients with well-controlled (<6.5%; left panel) or poorly controlled (≥8%) HbA1c eTable 7. Comparison of Type 2 Diabetes Remission Rates at 1, 3, and 5 Years across Subgroups eTable 8. Estimated percentage of T2DM remission by type of procedure and DiaRem Score eAppendix 2. Comparing Adjustable Gastric Banding to Roux-en-Y Gastric Bypass and Sleeve Gastrectomy [file jamasurg-e200087-s001.pdf]
